# Oxide-silicate petrology and geochemistry of subducted hydrous ultramafic rocks beyond antigorite dehydration (Central Alps, Switzerland)

**DOI:** 10.1007/s00410-023-02032-w

**Published:** 2023-08-16

**Authors:** Joana Filipa Vieira Duarte, Thomas Pettke, Jörg Hermann, Francesca Piccoli

**Affiliations:** Institute of Geological Sciences, Baltzerstrasse 1+3, 3012 Bern, Switzerland

**Keywords:** Metaperidotite, Redox, Subduction, Oxide, Silicate, Geochemistry

## Abstract

Oxide minerals contained in ultramafic rocks are useful tools to assess the redox conditions of the rock and fluids liberated upon progressive serpentinite dehydration during subduction, as these minerals contain a relevant redox-sensitive element, iron. Previous studies have revealed that magnetite predominates across the antigorite-out reaction. However, the fate of magnetite and other oxides at higher pressure and temperature conditions has remained underexplored. We present a comprehensive petrological and geochemical study of oxide-sulfide-silicate mineral assemblages in metaperidotites beyond antigorite- and chlorite-out reactions (T = 650–850 °C and P = 1–3 GPa). Several ultramafic lenses, covering different bulk rock compositions and extents of oxidation upon oceanic serpentinization, were investigated from the Central Alps, Switzerland. Results point to two endmember scenarios: (i) Most frequently, metaperidotites have olivine with a Mg# of 89–91 (defined as molar Mg/(Mg + Fe_tot_) × 100) and contain low oxide modes (0.06–1.41 vol.%), hematite is absent, and redox conditions are weakly oxidized and buffered by orthopyroxene-olivine-magnetite. (ii) Rare occurrence, high olivine Mg# > 94.5 metaperidotites display coexisting hematite and magnetite, high oxide modes (up to 4 vol.%), and redox conditions are hematite-magnetite (HM) buffered (Δlog_10_fO_2_,_QFM_ of + 3 to + 4). Spinel displays evolving compositions from magnetite over chromite to Al-Cr-spinel, roughly correlating with increasing temperature. Most of the samples buffered by the olivine-orthopyroxene-magnetite assemblage contain coexisting pentlandite ± pyrrhotite, thus identifying stable sulfides beyond antigorite dehydration for these weakly oxidized samples (Δlog_10_fO_2_,_QFM_ < 2.5). No sulfides were recognized in the highly oxidized sample. The transition of magnetite to chromite at around 700 °C goes along with a shift in fO_2_ to lower values. At the prevailing oxygen fugacity in the weakly oxidized metaperidotites sulfur in a coexisting fluid is always present in its reduced form. However, oxidized sulfur can be stable in the dehydration fluids released from highly oxidized serpentinites.

**Supplementary Information:**

The online version contains supplementary material available at 10.1007/s00410-023-02032-w.

## Introduction

Serpentinites are important carriers of redox budget into subduction zones, as they contain ferric iron in magnetite, serpentine minerals, chlorite, sometimes along with some carbonates (e.g., Evans 2012; Klein et al 2014). Redox budget is a mass-dependent parameter that describes the oxidizing capacity of a rock–fluid system, defined by the number of moles of electrons that need to be added or removed from the system to reach the reference state (Evans 2006). In particular, the stability of magnetite in ultramafic rocks plays a major role in controlling the redox state and the redox budget of the fluids liberated upon progressive dehydration reactions, as well as of the residual rocks during subduction (e.g., Piccoli et al 2019; Vieira Duarte et al 2021; Evans and Frost 2021). The principal dehydration reactions during subduction at elevated pressures are the brucite-out, antigorite-out (Eq. 1), and chlorite-out (e.g., Eqs. 2.1 and 2.2) reactions, releasing 0–3, 5–12, and 2.5–3 wt.% H_2_O, respectively (Ulmer and Trommsdorff 1995; Padrón-Navarta et al 2013; Lakey and Hermann 2022):

1 $${\text{Antigorite }} = {\text{ Olivine }} + {\text{ Orthopyroxene }} + {\text{ Chlorite }} \pm {\text{ Magnetite }} + {\text{ H}}_{{2}} {\text{O,}}$$

2.1 $${\text{Chlorite }} + {\text{ Orthopyroxene }} = {\text{ Garnet }} + {\text{ Olivine }} + {\text{ H}}_{{2}} {\text{O,}}$$

2.2 $${\text{Chlorite }} = {\text{ Spinel }} + {\text{ Olivine}} + {\text{ Orthopyroxene }} + {\text{ H}}_{{2}} {\text{O}}{.}$$

Depending on the fraction of Fe^3+^ in antigorite, the antigorite-out reaction (Eq. 1) can lead to the production of magnetite (Vieira Duarte et al 2021). Previous studies have suggested that upon antigorite dehydration during subduction, magnetite breaks down and significant redox budget is transferred to the fluids via oxidation of sulfides to produce fluid-mobile sulfate species (Debret et al 2015; Evans et al 2017; Merkulova et al 2017; Evans and Frost 2021). By contrast, evidence for production of only moderately oxidized fluids with fluid-mobile H_2_S at rock-buffered conditions (+ 1 < Δlog_10_fO_2_,_QFM_ <  + 2) has been presented more recently, as documented by (i) the coexistence of sulfide minerals such as pyrrhotite and pentlandite with magnetite (e.g., Vieira Duarte et al 2021); (ii) equilibrium thermodynamic modeling based on data from natural rocks (Piccoli et al 2019; Lazar 2020; Evans and Frost 2021); and (iii) dehydration experiments (Iacovino et al 2020). Moreover, it is demonstrated that upon antigorite dehydration magnetite is not only passively preserved but it actually recrystallizes, and new magnetite can form in equilibrium with prograde silicates, as documented for the chlorite-peridotites from Cerro del Almirez (Vieira Duarte et al 2021). Recently, Evans and Frost (2021) have reconciled these two scenarios based on closed-system thermodynamic modeling by suggesting that the redox budget of the serpentinite protoliths is the controlling factor on whether aqueous sulfide or sulfate species may predominate in dehydration fluids.

Evans and Frost (2021) modeled the evolution of two starting compositions with progressive subduction, (i) moderately oxidized serpentinites (reactive bulk Mg# of ~ 91) and (ii) strongly oxidized serpentinites (reactive bulk Mg# of ~ 96). Their closed-system model results indicate that magnetite is stable in strongly oxidized hydrous metaperidotites up to temperatures of 850 °C but lost in moderately oxidized hydrous metaperidotites at temperatures exceeding 700 °C, i.e., beyond antigorite dehydration. These authors also suggested that in the presence of magnetite, oxidized sulfur species can be mobilized in dehydration fluids at Δlog_10_fO_2_,_QFM_ of ca. + 2.5 at temperatures and pressures above 650 °C and 2 GPa, respectively. These conditions encompass both the antigorite- and chlorite-out reactions. However, the fate of the oxide and sulfide minerals beyond antigorite dehydration in natural samples has remained poorly constrained to date. Exceptions are scattered reports of coexisting magnetite, chromite, spinel, and sulfides in a few localities from the central European Alps (e.g., Piccoli et al 2019; Pfiffner 1999; Trommsdorff and Evans 1969), besides those from Cerro del Almirez, Spain (Trommsdorff et al 1998; Vieira-Duarte et al. 2021).

This study provides an assessment of the peak metamorphic oxide-silicate-sulfide mineral assemblages for the pressure and temperature window covered from antigorite-out to beyond chlorite-out reactions (approximately between 650 to 850 °C and 1–3 GPa) for a series of ultramafic bodies from the central European Alps, Switzerland. Our comprehensive petrological and geochemical study of oxide-silicate-sulfide mineral assemblages includes major element characterization of silicates and sulfides (EPMA) and major to trace element data for oxides (EPMA, LA-ICP-MS). We aim at documenting the range in oxidation covered by subducted oceanic serpentinites from plate interface domains. With this we aim at the better understanding of the driving forces of redox budget variations in hydrous metaperidotites, and its evolution during progressive subduction. Our focus is on the conditions required for the potential release of oxidized or reduced fluids from dehydrating serpentinites that may mobilize prominent redox budget as aqueous sulfate species, possibly ultimately reaching the loci of partial melting in the mantle wedge to generate comparatively oxidized arc magmas.

### Geological setting

**Fig. 1:**
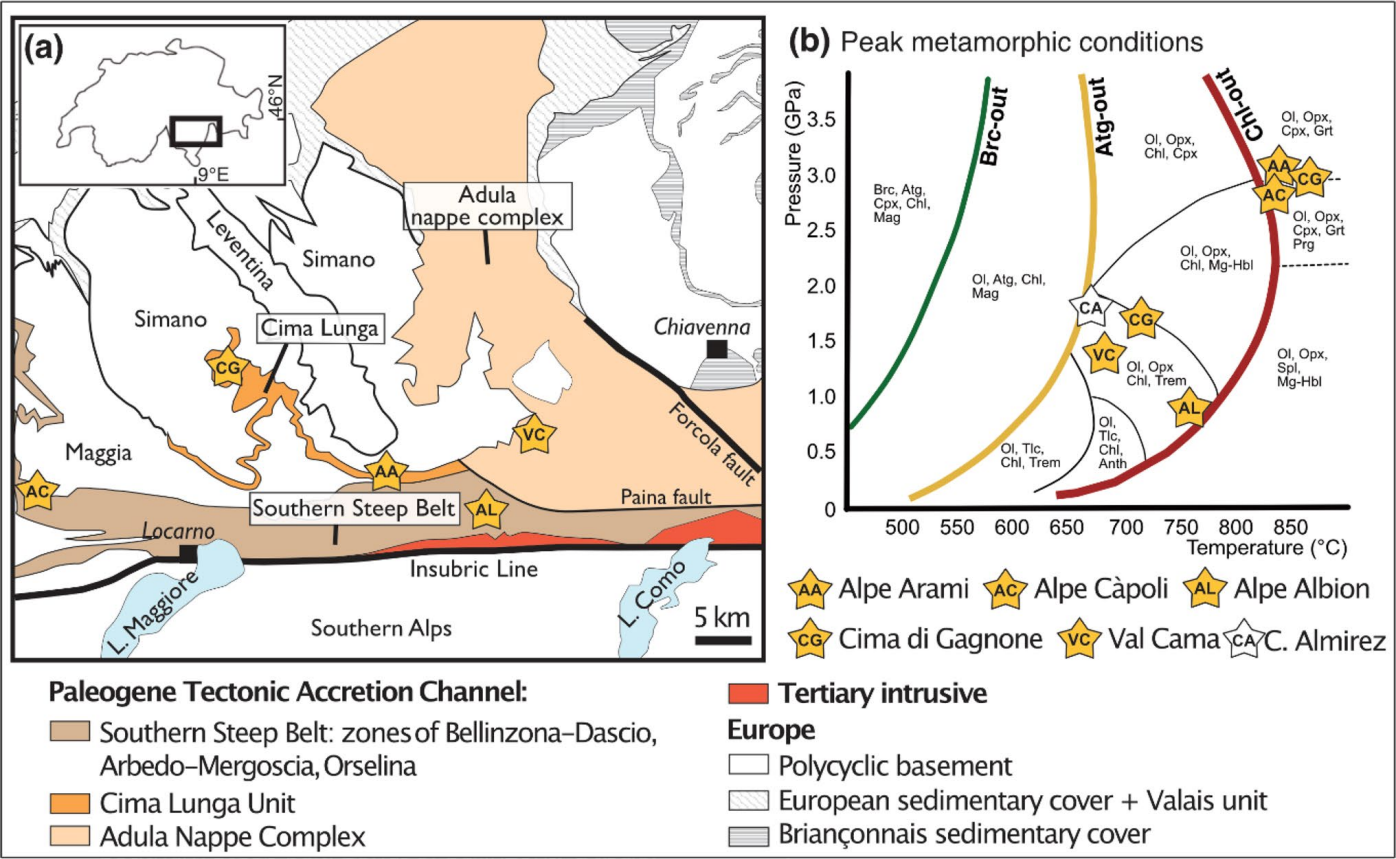
Geological setting of the studied ultramafic bodies of the Central Alps (Switzerland). **a** Tectonic sketch of the main geological nappes of the Central Alps (adapted from Piccoli et al. 2021). **b** Peak temperature conditions achieved by the studied hydrous ultramafic bodies expressed in a generic pressure–temperature diagram. The two stars for Cima di Gagnone (CG) likely represent different peak pressures achieved by two different bodies as suggested by different amphibole compositions. Peak conditions of Cerro del Almirez (Spain; Bretscher et al. 2018) are also plotted for comparison. Mineral abbreviations after Whitney and Evans (2010)

Hydrous metaperidotite bodies of the central European Alps occur scattered throughout the Southern Steep Belt in the Bellinzona-Dascio Zone and Adula-Cima Lunga unit (Fig. 1a). These units represent the subduction plate interface and consist of a lithospheric mélange (Engi et al 2001) where meter to kilometer-sized ultramafic bodies associated with metabasalts and locally calcite marbles (i.e., the former oceanic lithosphere) are embedded in highly foliated ortho- and paragneisses and associated migmatites (Berger et al 2005). Variable extents of rodingitization of metabasalts (e.g., Evans et al 1981) demonstrate oceanic hydration and metasomatism of the former oceanic lithosphere. Subduction metamorphic overprint invariably exceeded antigorite dehydration conditions in metaperidotites, most commonly forming chlorite-peridotites with well-equilibrated oxide-sulfide-silicate mineral parageneses recording peak temperature conditions. Peak pressure–temperature conditions range between ca. 650–850 °C and 0.8–3 GPa, respectively (e.g., Heinrich 1986; Nimis and Trommsdorff 2001; Trommsdorff and Evans 1972), enabling to explore a large range of the *P–T* space between antigorite- and chlorite-out reactions (Fig. 1b). Our sample set comprises hydrous metaperidotites from the Alpe Albion, Val Cama, Alpe Arami, Alpe Capoli, and Cima di Gagnone (Fig. 1; sample coordinates available in the Supplementary Table S1), and the lithological rock names refer to the respective peak metamorphic mineral assemblages (see discussion below and Table 1).

**Table 1:** List of samples studied in this work, organized by their metamorphic lithotypes. Sampling localities, mineral assemblages, and estimated modes for silicate and oxide minerals are also indicated

Peak metamorphic lithology	Sample	Locality	Mineral Assemblages		Modes of silicate minerals (vol.%)						Oxide mode (vol.%)				Sulfide mode (vol.%)
			Peak temperature	Retrograde	Ol	Opx	Cpx^1^	Chl	Am	Grt	Total	Fe-Cr	Al	Fe-Ti	
Hem-Mag-Chl-peridotite	PkCa-06	Val Cama	Ol, Opx, Chl, **Hem**, **Mag**	Mg-Ath	60	30		7			2.67	2.62	–	0.05	0.00
Mag-Chl-peridotite	Alb18-10	Alpe Albion	Ol, Opx, Chl, Trem, **Mag**	Mg-Ath	87	2		7			3.58	3.57	0.01	0.00	0.00
	Alb18-11	Alpe Albion	Ol, Opx, Chl, **Mag**		55	29		14			2.38	2.38	–	0.00	0.00
	Alb18-14	Alpe Albion	Ol, Opx, Chl, Trem, **Mag**		43	45		8.5	2		1.73	1.73	–	0.00	0.00
	Alb18-12	Alpe Albion	Ol, Opx, Chl, **Mag**		52	38		9	?		0.74	0.74	–	0.00	0.00
	Alb18-09	Alpe Albion	Ol, Opx, Chl, Trem, **Mag**	Mg-Ath	54	34		8	3		0.65	0.65	–	0.00	0.00
	CdG19-50	C. di Gagnone	Ol, Opx, Chl, Trem, **Mag**, **Ilm-Hem**		57	33		8	1		0.55	0.44	–	0.11	0.00
	CdG19-37	C. di Gagnone	Ol, Opx, Chl, Trem, **Mag**	Mg-Ath	59	23		14	4		0.48	0.48	–	0.00	0.00
	PkCa-08	Val Cama	Ol, Opx, Chl, Trem **Mag**, **Ilm**		36	11		26	27		0.21	0.10	–	0.11	0.00
Chr-Chl-peridotite	Alb18-15	Alpe Albion	Ol, Opx, Chl, Mgs, Trem, **Chr**, Pn, Po		62	24		8	9		0.38	0.38	–	0.00	0.16
	PkCa-01	Val Cama	Ol, Opx, Chl, Trem, **Chr**, Pn	Mg-Ath	59	14		8	19		0.17	0.17	–	0.00	0.01
	Alb18-03	Alpe Albion	Ol, Opx, Chl, Mg-Hbl, **Chr**, **Spl**		49	36		13	3		0.11	0.11	–	0.00	0.00
Chr-peridotite	AR18-11	Alpe Arami	Ol, Opx, Mg-Hbl, **Chr**, Pn	Chl, Trem	73	11		8.5	7		0.27	0.27	–	0.00	0.01
	AR18-01	Alpe Arami	Ol, Opx, **Chr**	Chl	94	3		2.5			0.27	0.27	–	0.00	0.00
	AR18-07	Alpe Arami	Ol, Opx, Mg-Hbl, **Chr**, Pn, Hzl	Chl, Trem	78	5		11	9		0.19	0.19	–	0.00	0.01
	Cap18-01	Alpe Capoli	Ol, Opx, **Chr**, Pn, Hzl	Chl, Trem	53	16	2.5	14	15		0.11	0.11	–	0.00	0.00
	AR20-01	Alpe Arami	Ol, Opx, **Chr**, Pn	Chl	41	37		25	1		0.10	0.10	–	0.00	0.05
Grt-peridotite	Cap18-03	Alpe Capoli	Ol, Opx, Cpx, Hbl, **Mag**,** Ilm-Hem**	Trem, Spl	40	43	6		10		1.31	0.82	0.10	0.39	0.00
	CP16-10	Alpe Capoli	Ol, Opx, Cpx, Hbl, **Chr**, **Ilm**	Trem, Spl	80	10	7		3		0.18	0.16	n.d	0.02	0.00
	AR20-02b	Alpe Arami	Ol, Opx, Cpx, Grt, Hbl, **Chr**, Pn		57	26	8		2	7	0.11	0.11	n.d	0.00	0.01
	Mg160-96–1^2^	C. di Gagnone	Ol, Opx, Cpx, Grt, Hbl, **Ilm**, Pn	Spl	58	11	9		2	20	0.07	0.06	–	0.01	0.01
	AR18-10	Alpe Arami	Ol, Opx, Cpx, Grt, Hbl, **Chr**, **Ilm**, Pn, Po		59	21	6		1	13	0.06	0.05	n.d	0.01	0.04
	CdG19-32	C. di Gagnone	Ol, Opx, Cpx, Grt, **Chr**	Trem	53	16	12		x	20	0.04	0.04	n.d	0.00	0.00

Silicates modes were roughly estimated by transmitted light microscopy and adjusted relative to bulk rock data, while oxide and sulfide mineral modes were more precisely estimated by image analysis of reflected light scans acquired with MIA Scan. Minor retrograde lizardite/chrysotile and talc were found in most samples

x Observed but in trace amounts, making it difficult to estimate the modes

^1^Difficult to estimate petrographically

^2^Silicate mineral modes from Scambelluri et al. (2014)

### Moderate pressure–temperature samples: Val Cama and Alpe Albion

Metaperidotites from Val Cama and Alpe Albion equilibrated at temperature conditions situated between the antigorite-out (Eq. 1; Fig. 1b) and the chlorite-out (Eqs. 2.1 and 2.2) reactions at moderate pressures. Peak temperature and pressure conditions at Val Cama were estimated at ~ 660 °C and ~ 1.4 GPa based on thermodynamic calculations on garnet-chlorite-spinel in amphibolites (Dale and Holland 2003), and on anorthite-calcite and diopside-calcite-quartz in adjacent marbles (Trommsdorff and Evans 1969). Former oceanic hydration and metasomatism of the metaperidotites is confirmed by metamorphic olivine and orthopyroxene highly enriched in B, up to 100- and 10-times primitive mantle concentrations (Reichenwallner 2015). For Alpe Albion, minimum temperature estimates are derived from the stability of tremolite-hornblende at ca. 700–750 °C (this study) at peak pressures of 0.5–0.8 GPa (Schmidt 1989).

### High pressure–high temperature samples: Alpe Arami, Alpe Capoli, and Cima di Gagnone

Metaperidotites from Alpe Arami, Cima di Gagnone, and Alpe Capoli at least in part reached the highest peak temperature and pressure conditions across the chlorite-out reaction (Eqs. 2.1 and 2.2, Fig. 1b). Alpe Arami and Cima di Gagnone are famous for the occurrence of garnet-peridotites, while chlorite-peridotites dominate at Alpe Capoli. Alpe Capoli hosts retrogressed garnet-peridotites (see discussion below). Peak temperature and pressure conditions in garnet-peridotites and associated rocks were estimated to 750–850 °C and 2.5–3 GPa at Cima di Gagnone (Evans and Trommsdorff 1978; Pfiffner 1999; Nimis and Trommsdorff 2001; Scambelluri et al 2014; Piccoli et al 2021), ca. 800 °C and 3 GPa at Alpe Capoli (Lederer 2019), and to ca. 840 °C and 3.2 GPa in Alpe Arami (Nimis and Trommsdorff 2001; Buholzer 2020). The metamorphic conditions of chlorite-peridotites are less constrained (see below). Former oceanic hydration and metasomatism of the metaperidotites are confirmed for Cima di Gagnone by abundant rodingites (Evans and Trommsdorff 1978) and trace element systematics in bulk rocks and peak metamorphic silicate minerals (Scambelluri et al 2014).

## Methods

Hydrous metaperidotite bodies sampled in the five localities were selected to cover the large compositional variability of each body (3 samples from Alpe Cama, 7 from Alpe Albion, 6 from Alpe Arami, 4 from Cima di Gagnone, and 3 from Alpe Capoli). A detailed petrography of each sample was performed by transmitted and reflected light microscopy, scanning electron microscopy, and Raman spectroscopy. Opaque mineral modes were estimated by image processing of high-resolution reflected light scans obtained by a multiple image alignment approach as employed in Vieira Duarte et al (2021). Peak silicate modes were estimated by less precise image analysis of transmitted light scans. Modes were adjusted by combining mineral major element compositions together with bulk rock data. By this procedure, maximum chlorite modes were estimated by aluminum contents in the bulk, and olivine and orthopyroxene modes recalculated proportionally.

### Bulk rock measurements

Bulk rock chemical analyses were conducted for 18 samples covering the different lithologies from Alpe Cama, Alpe Albion, Alpe Arami, and Alpe Capoli. Weathered rock surfaces were first removed using a bench vice equipped with stainless-steel chisel heads. Rock milling (all in agate) and the production of pressed powder pellets (PPP) for LA-ICP-MS measurements employed the procedures detailed in Peters and Pettke (2017). Fifty-eight elements were recorded during LA-ICP-MS analysis, using a GeoLas-Pro 193 nm ArF Excimer laser system (Lambda Physik), coupled with an ELAN DRC-e quadrupole mass spectrometer (Perkin Elmer). Laser ablation was performed using an energy density of ~ 8 J cm^−2^, a repetition rate of 10 Hz, and a beam size of 120 µm. The surface was cleaned by pre-ablation using a larger beam size. Each PPP was measured on 6 individual spots, and the results reported represent the average and its 1 standard deviation measurement uncertainty. The trace element doped basaltic glass GSD-1G was used as the external calibration standard and for linear drift correction (preferred values listed by Peters and Pettke 2017). Data reduction was performed using the SILLS software (Guillong et al 2008), and limits of detection were calculated with the method of Pettke et al (2012). Internal standardization was done by normalizing the measurement data to a fixed total of 100 wt.% major element oxides (SiO_2_, MgO, FeO, Al_2_O_3_, TiO_2_, MnO, CaO, Na_2_O, K_2_O, and P_2_O_5_) minus water as determined by loss on ignition (carbonates were insignificant in our samples). Results for iron are reported as FeO_tot_, due to lack of measurement data for ferric iron in the bulk rock samples. Measurement accuracy was monitored by using a PPP of the OKUM standard produced in the same way as the sample PPP. Major element concentrations measurements for SiO_2_, MgO, FeO_tot_, Al_2_O_3_, TiO_2_, MnO, CaO, and Na_2_O deviate less than 5%, and Mg# less than 0.5, from reference values (compiled in Peters and Pettke 2017).

Average compositions per sample are provided in the Supplementary Tables S2–S10, and the full data set is available at Zenodo repository at https://doi.org/10.5281/zenodo.7516492.

### Major element analysis

Mineral chemistry of silicate minerals was determined by WDS using a JEOL JXA 8200 superprobe at the Institute of Geological Sciences of the University of Bern, operating with an acceleration voltage of 15 keV, a probe current of 10 nA, and a beam diameter of 1 μm. Spot analyses were measured for each mineral phase present. The mass fractions of 8 element oxides were calibrated using synthetic and natural standards. High-resolution maps were acquired using the wavelength dispersive spectrometers (WDS) and point analyses serving as internal standards (Lanari and Piccoli 2020). Analytical conditions included acceleration voltage of 15 keV and probe current of 100 nA to compensate for the short dwell times (Lanari and Piccoli 2020). X-ray maps were corrected for dead time, classified, and standardized using XMapTools 4 (Lanari et al. 2014, 2019). Different measurement settings and standards were employed for silicate, oxide, and sulfide minerals, which is described in detail in Supplementary Information S1.

### Trace element analysis

Minor to trace element compositions were measured in selected oxides by LA-ICP-MS, employing a Resonetics RESOlution SE 193 nm excimer laser system equipped with an S-155 large volume constant geometry chamber (Laurin Technic, Australia) coupled to an Agilent 7900 quadrupole ICP-MS instrument. Tuning and measuring conditions followed the routine employed at Vieira Duarte et al (2021). The beam size was set to between 16 and 30 µm, depending on the grain size, and the surface area of each measurement spot was cleaned by pre-ablation for three pulses employing a slightly larger beam size. Data reduction was performed by using the software Iolite (Hellstrom et al 2008; Paton et al 2011), applying a step-forward spline-type function fit between standard measurement spots. External standardization was done with the doped basaltic glass GSD-1G and by internal standardization to 100 wt% element oxides (MgO, FeO, Cr_2_O_3_, Al_2_O_3_, TiO_2_, MnO, NiO, and ZnO normalized to 100% (m/m); Beta_TE_Norm reduction scheme in Iolite Igor Pro, version 7.08). Measurement accuracy was tested for by measuring the magnetite standard BC-28 (Bushveld complex) and the glass standard SRM612 from NIST during each session.

For standard BC-28, variations in apparent measurement accuracy between sessions were registered in Ti, V, and Mn of ca. 20% in one measurement session; during this session, measurement data for SRM612 varied by less than ca. 4%, thus indicating heterogeneity in BC-28 for Ti, V, and Mn. Averaged concentrations measured for BC-28 match the house working values provided in Barnes et al (2004), Dare et al (2012), and the Barnes and Savard LabMaTer-UQAC in-house compilation of 2017. Minor to trace elements of minerals of the ilmeno-hematite solid-solution minerals were measured by LA-ICP-MS in three samples and using a large beam size (20 µm, much larger than the ~ 2 µm exsolution lamellae) to obtain their bulk composition prior to retrograde exsolution.

## Results

### Petrography

Table 1 lists the samples investigated along with their mineral modes. Based on petrographic observations, two groups of metaperidotites can be distinguished: those containing peak chlorite (chlorite-peridotites) and those containing chromite or garnet as the peak metamorphic aluminum-bearing phase (chromite-peridotites and garnet-peridotites). Magmatic relic minerals were never observed. Peak metamorphic rock names were derived based on the index Fe-Cr oxide and silicate minerals stable at peak temperature conditions.

**Fig. 2:**
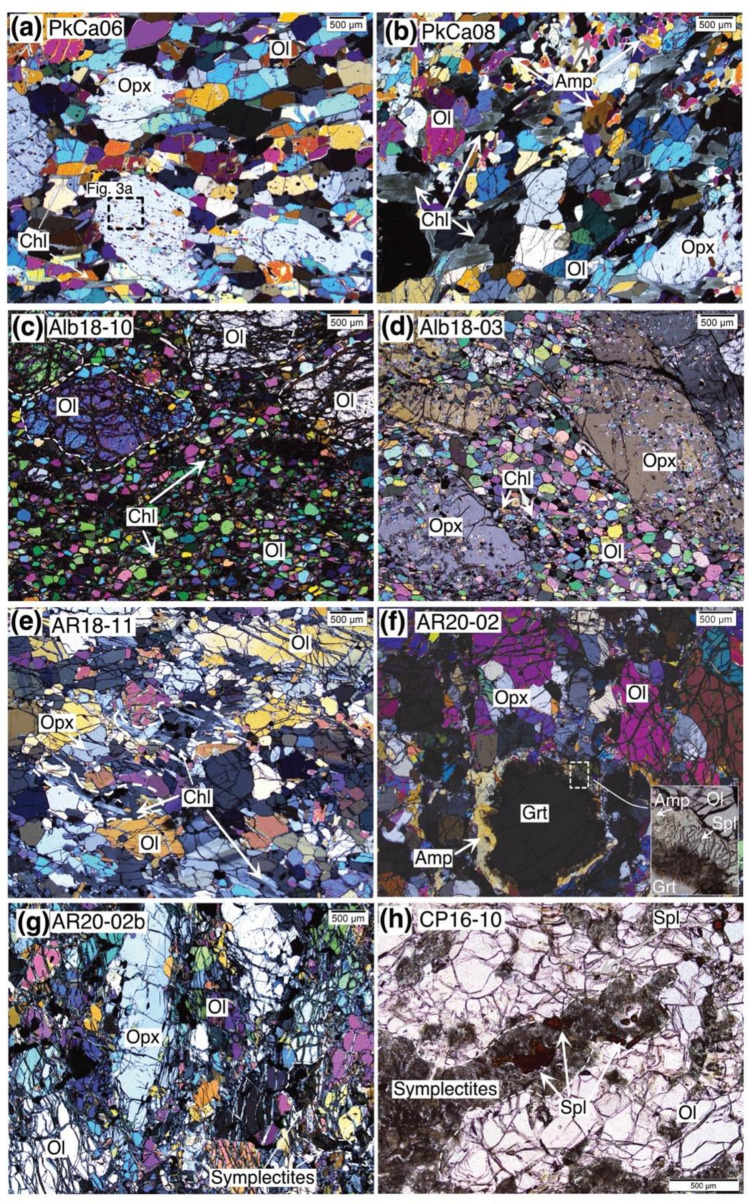
Microphotographs of different metamorphic lithotypes found in samples from the studied localities. All images were acquired in cross polarized transmitted light microscopy to better visualize the texture and silicate minerals present, except **h** and inset in **f** that were acquired in plane polarized transmitted light microscopy. **a** Hem-Mag-Chl-peridotite showing perfect 120° grain boundaries between minerals; **b** and** c** Mag-Chl-peridotites showing polygonal granular textures with **b** local enrichments in amphibole, or **c** a dunite domain within the harzburgite. It is noted that only in sample Alb18-10, olivine is observed as large anhedral grains of ca. 1 mm, or small polygonal grains of ca. 150–200 µm. **d** Chr-Chl-peridotite showing polygonal granular textures with orthopyroxene forming euhedral grains with local olivine inclusions. **e** Chr-peridotite with granular texture, forming deformed domains with chlorite + chromite pockets; **f** Grt-peridotite forming granular textures. Symplectites of amphibole + spinel are observed along the rims of garnet. **g** and** h** Former Grt-peridotites showing granular textures with deformed domains of symplectites + chromite. Mineral abbreviations after Whitney and Evans (2010)

Chl-peridotites are widespread in all the studied localities and consist of polygonal to lobular granoblastic equilibrium textures (Fig. 2a–d). These rocks are composed of a mosaic of olivine (~ 100–500 µm size), interstitial chlorite laths (~ 200 µm) and unoriented orthopyroxene porphyroblasts (500 µm to 1.5 mm). Moreover, centimeter-sized oxide porphyroblasts (500 µm to 1.5 mm) are embedded and aligned along the foliation. Anhedral to prismatic amphibole (up to 1 mm) is observed in some samples but is generally minor (< 4 vol.%). Different oxide types can be recognized, thus defining three distinct sub-lithotypes: (i) chlorite-peridotite with magnetite and hematite (hereafter, Hem-Mag-Chl-peridotite; Fig. 2a); (ii) chlorite-peridotite with magnetite (hereafter, Mag-Chl-peridotite; Fig. 2b,c); and (iii) chlorite-peridotite with chromite (hereafter, Chr-Chl-peridotite; Fig. 2d). Ilmenite and ilmeno-hematite displaying retrograde exsolution textures (compare Vieira Duarte et al 2021) also occur. Hem-Mag-Chl-peridotites are represented by only one sample from Val Cama, showing the highest (Fe-Cr) oxide content (2.6–4 vol.%; this work; Trommsdorff and Evans 1969), and no sulfides. Mag-Chl-peridotites and Chr-Chl-peridotites are common across the Central Alps (e.g., Dal Vesco 1953; Trommsdorff and Evans 1969; Pfiffner 1999; this work). Mag-Chl-peridotites show variable (Fe, Cr) oxide modes between 0.7 and 3.6 vol.%, and sulfides were not observed. Chr-Chl-peridotites generally display low (Fe, Cr) oxide modes, between 0.11 and 0.38 vol.%, along with < 0.16 vol.% sulfide. In Chr-Chl-peridotites, chlorite can also occur as aggregates forming elongated to rounded domains of about 500 µm, often with oxides in the center (not shown).

Chromite-peridotites (hereafter, Chr-peridotites) were found exclusively at Alpe Arami and show a granoblastic texture similar to Chr-Chl-peridotites (Fig. 2e), except that chlorite in these samples occurs mainly along interconnected clusters or fractures, thus interpreted as a retrograde feature (see below). Chromite-peridotites show low (Fe-Cr) oxide modes, between 0.10 and 0.27 vol.%, and sulfide contents < 0.05 vol.%.

Garnet-peridotites (hereafter, Grt-peridotites), or relics thereof, occur at Alpe Arami, Cima di Gagnone, and Alpe Capoli. They show a granoblastic lobate texture (Fig. 2f–h), often preserving a foliation marked by elongated olivine, orthopyroxene, and clinopyroxene (~ 50–500 µm), as well as by the alignment of garnet porphyroblasts (up to 1 cm). Garnet-peridotites show generally low (Fe-Cr) oxide (< 0.16 vol.%) and sulfide (< 0.04 vol.%) contents, with exception of sample Cap18-03 with higher (Fe-Cr) oxide content (0.82 vol.%) and no sulfides.

Different amphibole types are variably present in the metaperidotites (Table 1) and include tremolite, Mg-hornblende to hornblende, and anthophyllite in one sample (Alb18-10).

### Oxide mineralogy

Magnetite, chromite, and spinel are the principal oxide phases found in the different lithologies. One sample contains hematite and magnetite in textural equilibrium with the peak silicate parageneses. Peak metamorphic ilmenite in textural equilibrium with the peak silicate parageneses and subordinate coexisting ilmenite and hematite exsolution lamellae after peak metamorphic ilmeno-hematite are sometimes also present. The different oxide phases identified in every sample, as well as their modes, are listed in Table 1.

### Hem-Mag-Chl-peridotite

**Fig. 3:**
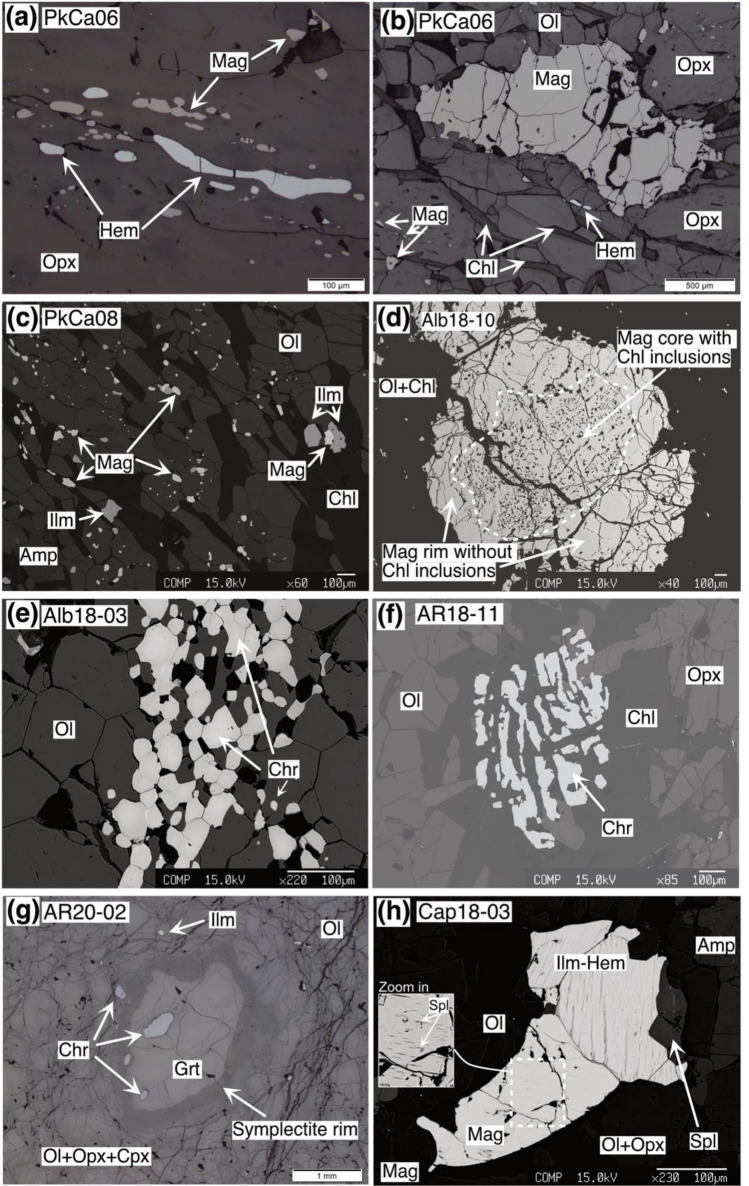
Microphotographs of the oxide minerals present in the different metaperidotites. Images **a**, **b**, and **g** were acquired by reflected light microscopy, the others by backscatter electron microscopy. **a, b** Hem-Mag-Chl-peridotite, showing in **a** inclusions of hematite and magnetite in orthopyroxene porphyroblasts; and in **b** interstitial hematite coexisting with orthopyroxene, chlorite, and magnetite. **c, d** Mag-Chl-peridotite, in **c** showing inclusions of magnetite and ilmenite in olivine and chlorite, and in **d** anhedral grains with Chl-rich inclusions in the core and inclusion-free rims. **e** Chr-Chl-peridotite, showing polygonal chromite inclusions in olivine. **f** Chr-peridotite with skeletal chromite in the center of chlorite aggregate. **g,h** Grt-peridotite showing in **g** chromite and ilmenite inclusions in garnet and olivine, and in **h** intergrowths of magnetite, spinel and ilmeno-hematite, magnetite also showing retrograde spinel exsolutions

Hematite and magnetite occur as inclusions (10–300 µm size) in peak metamorphic orthopyroxene (Fig. 3a) and olivine in sample PkCa-06, generally elongated and aligned within the large orthopyroxene porphyroblasts mimicking a prograde foliation. However, magnetite (up to 5 mm) and hematite (up to 100 µm) can also be found as inclusion-free subhedral grains at interstitial position and in equilibrium with the peak silicates (Fig. 3b). Considering the total amount of oxides in sample PkCa-06, the magnetite fraction is > 98 vol.%, whereas the hematite fraction is < 2 vol.%.

### Mag-Chl-peridotite

Magnetite is found sometimes aligned along the foliation in two different textural positions: (i) as anhedral inclusions (up to 100 µm size) in peak silicate minerals olivine, orthopyroxene, and chlorite (Fig. 3c) and (ii) as interstitial subhedral grains (up to 10 mm size) in textural equilibrium with peak silicate minerals, i.e., sharing straight boundaries. The latter can form polygonal inclusion-free aggregates or consists of an inclusion-rich core surrounded by inclusion-free rim (Fig. 3d), where in some cases (e.g., Alb18-10), fine (< 10 µm) spinel lamellae can be seen, interpreted to represent crystallization upon retrogression (discussed below).

### Chr-Chl-peridotite and Chr-peridotite

Chromite grains in these lithologies occur in two textural positions. Polygonal aggregates of ca. 50 µm size, occur aligned either as inclusions in orthopyroxene porphyroblasts or at interstitial position in equilibrium with olivine, orthopyroxene, and chlorite (e.g., sample Alb18-03; Fig. 3d). In addition, rounded-anhedral or skeletal grains (ca. 500 µm) within chlorite clots (Fig. 3e) can also be recognized.

### Grt-peridotite

Chromite together with ilmenite is the main oxide phase present in the Grt-peridotite from Alpe Arami, Alpe Capoli, and Cima di Gagnone, occurring either as anhedral inclusions (up to 500 µm size) in silicate minerals, including olivine and garnet (Fig. 3f,g), or in interstitial position, sometimes also associated with retrograde symplectites (Fig. 2h). One former Grt-peridotite from Alpe Capoli (Cap18-03), however, shows magnetite and exsolved ilmeno-hematite grains, forming anhedral grains in association with olivine and orthopyroxene grains. They also show multi-oxide inclusions (ca. 150 µm) in orthopyroxene, where the limit between magnetite and ilmeno-hematite is sharp but between spinel and magnetite is irregular (Fig. 3h).

### Sulfide mineralogy

Pentlandite (50–200 µm) is the most common sulfide present. Pentlandite occurs at grain boundaries, forming intergrowths with chlorite, and as inclusion in peak silicate minerals. In some samples, pentlandite occurs together with minor pyrrhotite, as is the case of the observed inclusions in garnet (see Supplementary Figure S1).

### Retrogression features

Retrograde features, defined here to include all minerals formed after the metamorphic peak pressure, are visible across the different lithologies (see Table 1). These include formation of chlorite, tremolite, talc, and lizardite/chrysotile in clusters (50–300 µm size). These clusters are interstitially connected or distributed along fractures (Fig. 2e). Retrogression clusters are interpreted to form by interaction with fluids during exhumation and cooling. As an index mineral for the study of the chlorite-out reaction, it is important to define whether chlorite occurs in equilibrium at peak conditions or if it formed only during retrogression. An example is chlorite clusters surrounding chromite observed in Chr-peridotites from Alpe Arami, which were interpreted as retrograde (Fig. 3f). On the other hand, retrograde mineral assemblages can be useful indicators of mineral stability at higher P–T conditions. In fact, Grt-peridotites from Alpe Capoli show conspicuous patchy zones with dusty appearance (Fig. 2g, h; Supplementary Figure S2), which are composed of very fine symplectites of green spinel, amphibole, and sometimes clinopyroxene associated to brown spinel. These symplectitic assemblages resemble those formed around preserved garnet crystals in Grt-peridotite of Alpe Arami (Fig. 2g), thus suggesting former garnet stability. This textural evidence is supported by EDS scan estimates obtained by SEM on large areas of the symplectites, whose bulk compositions largely match garnet compositions (see Supplementary Figure S2). We therefore categorize these samples as garnet-peridotites, interpreting the symplectites to represent retrograde destabilization of garnet upon fluid infiltration.

Retrogression involving oxide and sulfide minerals is also recognized. Exsolution of ilmenite on hematite grains likely formed upon cooling after ilmeno-hematite solid solution minerals (compare Vieira Duarte et al 2021), and spinel exsolutions in magnetite (Fig. 3h) are some of the oxide textures formed during retrogression. Sulfides associated with retrogression features include rare occurrences of chalcopyrite (ca. 20 µm size) along late fractures, or oxides along cracks on pentlandite and pyrrhotite, both in areas filled with mesh-serpentine and talc.

### Bulk rock data

**Fig. 4:**
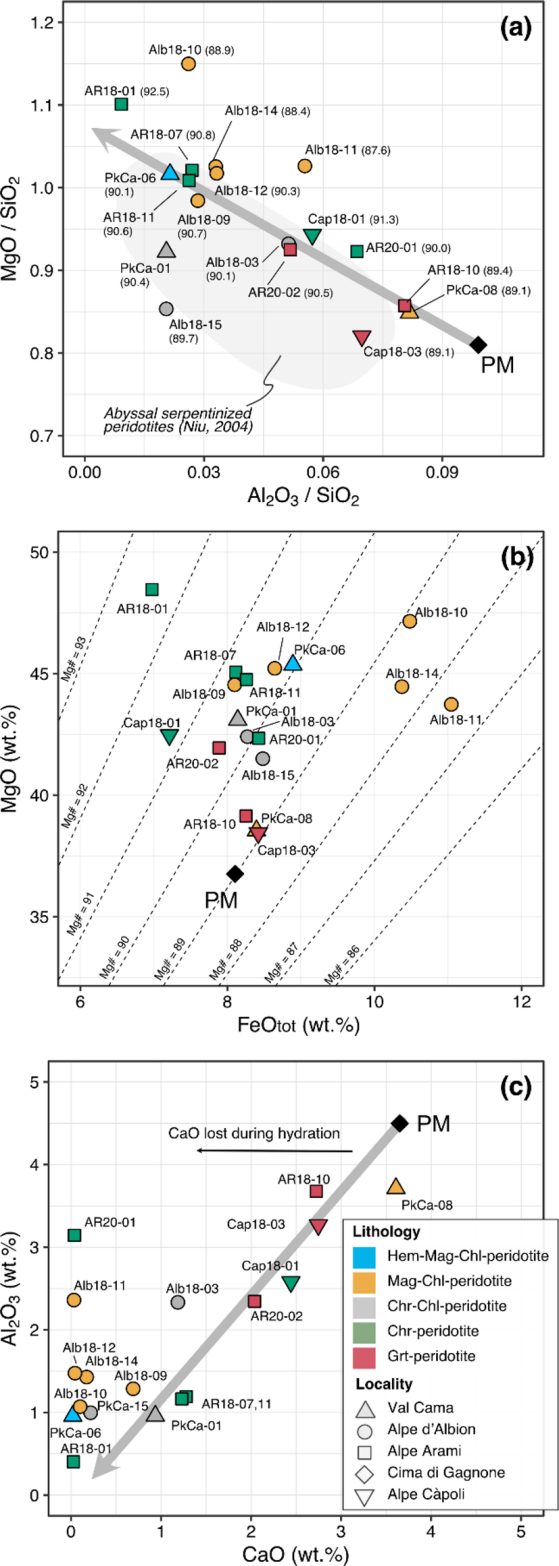
Bulk rock major element data for eclogite-facies metaperidotites. **a** MgO/SiO_2_ vs. Al_2_O_3_/SiO_2_. Sample labels (Table 1) are given with bulk rock Mg# (in brackets). The field of abyssal peridotites (Niu 2004) is shown for reference. **b** MgO vs. FeO_tot_, contoured with iso-Mg# lines. **c** Al_2_O_3_ vs. CaO. The thin black arrow displays the trend of CaO loss during serpentinization. Thick gray arrows in **a** and **c** show the residual peridotite trend after progressive melt extraction from primitive mantle (PM) composition (Niu 2004; Palme and O’Neill 2014). Symbol colors reported in **c** correspond to the lithologies reported in Table 1

Bulk rock major element compositions were investigated for 18 samples covering all lithologies. The rock sample suite displays variably depleted peridotite signatures relative to primitive mantle composition (PM; Palme and O’Neill 2014), including lherzolites, harzburgites, and more rarely dunites. This variability results in a spread along the melt extraction residue array (Niu 2004), as shown in the MgO/SiO_2_ vs. Al_2_O_3_/SiO_2_ plot (Fig. 4a). Samples plotting below this trend fall into the field for serpentinized abyssal peridotites (Niu 2004). Samples plotting distinctly above this trend (e.g., Alb18-10, Alb18-11) contain high olivine modes (Table 1) and low bulk rock Mg# (Fig. 4a; Mg# shown within brackets). Most samples display Mg# typical for oceanic peridotites ranging between 89.0 and 91.3, with one dunite sample (AR18-01) at 92.5 that does not correlate with bulk rock FeO_tot_ concentrations (Fig. 4b; all iron concentrations are reported as FeO_tot_, because there are no data available for the fraction of ferric iron in our samples). In a plot of Al_2_O_3_ vs. CaO (Fig. 4c), most of the samples depart from the melt depletion trend toward lower CaO for a given Al_2_O_3,_ which is indicative of serpentinization (e.g., Niu 2004; Paulick et al 2006). A few samples, however, show a relative enrichment in CaO for the respective Al_2_O_3_ concentration that correlates with elevated modes of clinopyroxene or tremolite.

### Major element mineral chemistry

#### Silicate minerals

**Fig. 5:**
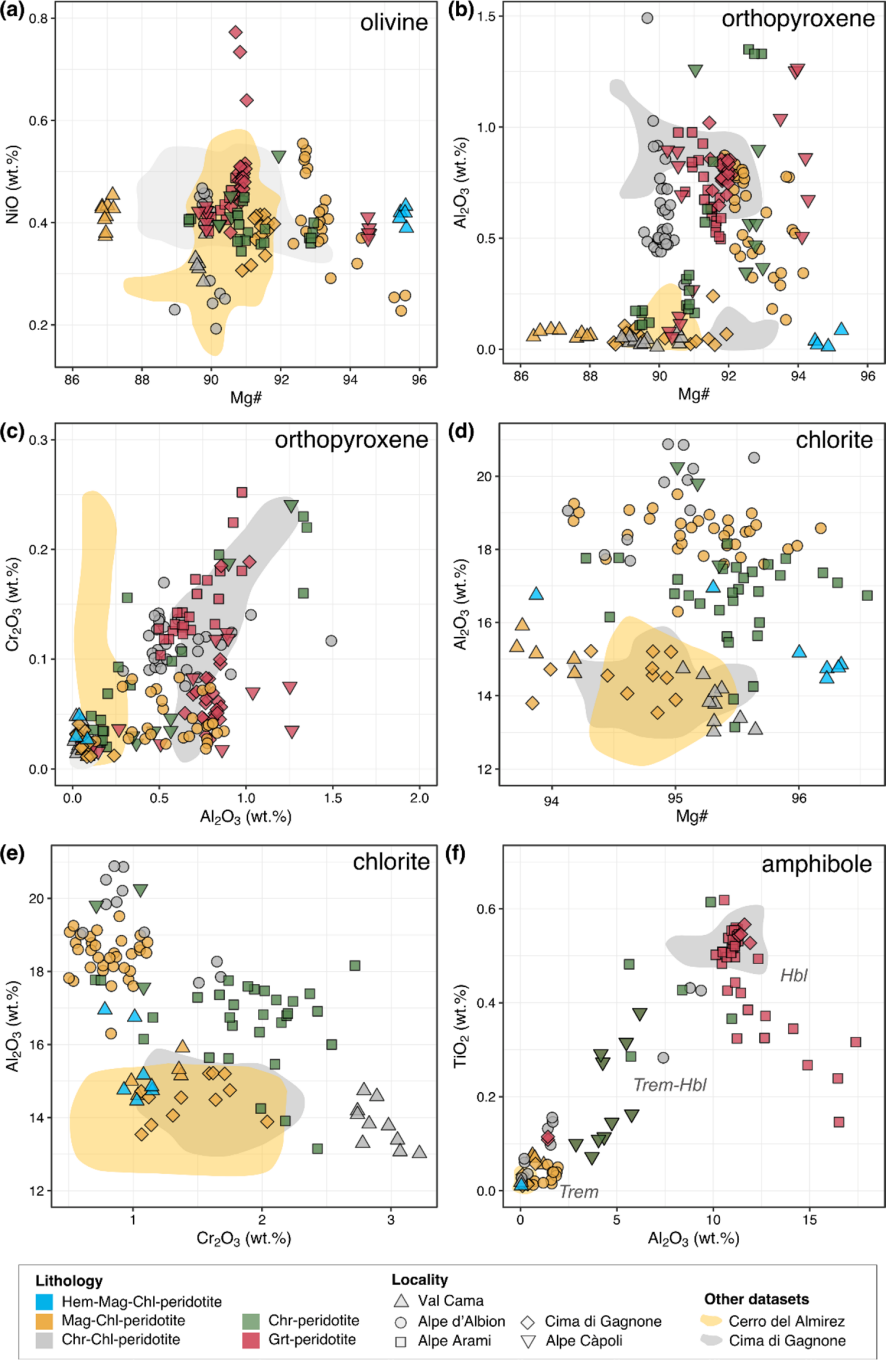
Major element silicate mineral chemistry of samples of this study compared to selected literature data, for **a** olivine; **b, c** orthopyroxene; **d, e** chlorite; and **f** amphibole. Data for Chl-peridotites of Cerro del Almirez are from Bretscher et al. (2018) and for Chl-peridotites and Grt-peridotites of Cima di Gagnone from Pfiffner (1999)

Silicate minerals are generally unzoned, but compositional variations are observed between samples. Major element concentration data of olivine, orthopyroxene, chlorite, and amphibole are displayed in Fig. 5 and in Tables S3–S8 provided in Supplementary Materials. Data are grouped by lithology as described above (Section Petrography) and compared to Chl-harzburgite and Grt-peridotite data from other well-known localities (Pfiffner 1999; Scambelluri et al 2014; Bretscher et al 2018).

The olivine composition in the samples covers a wide range in terms of Mg# from 87.0 to 95.6. The highest values are found in the Hem-Mag-Chl-peridotite and few grains in Mag-Chl-peridotite (Fig. 5a). Olivine in different Mag-Chl-peridotites samples also shows a wide range, which is generally shifted to high Mg# (91.4–95.6) except for sample PkCa-08 that has olivine with Mg# of 87.0. In contrast, ranges in olivine Mg# are narrower between 89.7 and 91.1 for Chr-Chl-peridotite, Chr-peridotite, and Grt-peridotite, except for sample Cap18-03 and AR18-01, with Mg# of 94.5 and 92.9, respectively. NiO contents are generally between 0.30 and 0.77 wt.%, except for some Chr-Chl-peridotites and one Mag-Chl-peridotite (Alb18-10), with lower values of 0.21–0.46 wt.% and 0.26–0.42 wt.%, respectively. Olivine compositions from this study generally overlap with those from Chl-harzburgites from Cerro del Almirez (Bretscher et al 2018) and Cima di Gagnone (Pfiffner 1999; Scambelluri et al 2014), with Mg# between 88.0 and 91.5. Nevertheless, NiO contents in Almirez Chl-peridotites show a larger range, namely between ~ 0.15 and 0.58 wt.%.

The range of orthopyroxene Mg# (86.9–95.2; Fig. 5b) is overlapping with that displayed by olivine. This is expected when metamorphic olivine and orthopyroxene coexist in equilibrium. Aluminum and chromium are variable from < 0.01 wt.% (detection limit) up to 1.5 and 0.25 wt.%, respectively, for Al_2_O_3_ and Cr_2_O_3_, reaching the highest Cr content in the Grt-peridotite and some Chr-peridotite (Fig. 5c). In contrast, Mag-Chl-peridotites all show lower Cr_2_O_3_ contents (< 0.1 wt.%). Orthopyroxene in Mag-Chl-peridotite, Chr-Chl-peridotite, and Chr-peridotite is generally lower in CaO (0.02–0.21 wt.%) when compared to that of Grt-peridotite (0.15–0.26 wt.%). Orthopyroxene compositions with low Al_2_O_3_ (< 0.26 wt.%) and Cr_2_O_3_ (< 0.15 wt.%) contents, but variable Mg#, resemble those reported for Chl-peridotites from Cerro del Almirez (Bretscher et al 2018) and Cima di Gagnone (Pfiffner 1999; Scambelluri et al 2014). However, orthopyroxene compositions that show higher Al_2_O_3_ (> 0.60 wt.%) and Cr_2_O_3_ (> 0.50 wt.%) contents are similar to Grt-peridotite from Cima di Gagnone (Pfiffner 1999; Scambelluri et al 2014). Clinopyroxene only occurs in the Grt-peridotites but shows a large variability between samples with Mg# of 92.5–94.9, variable Cr_2_O_3_ (~ 0.08–1.02 wt.%), Al_2_O_3_ (0.38–2.18 wt.%), and FeO_tot_ (1.61–2.38 wt.%).

Chlorite shows clinochlore compositions with variable Mg# (93.9–96.2), Al_2_O_3_ (13–21 wt.%), Cr_2_O_3_ (< 3.5 wt.%), and SiO_2_ (29–34 wt.%) contents, irrespective of the metamorphic lithotype (Fig. 5d). Its compositional range is much larger than concentrations reported for chlorite from Cerro del Almirez (Bretscher et al 2018) and Cima di Gagnone (Pfiffner 1999; Scambelluri et al 2014), with Al_2_O_3_ < 16 wt.% and Cr_2_O_3_ < 2.5 wt.%.

Garnet in the Grt-peridotites shows small variations, with X_Prp_ between 0.56 and 0.65, X_Alm_ between 0.13 and 0.19, and X_Grs_ between 0.19 and 0.25. Small differences between core and rim are also observed, for instance, in sample AR20-02b with X_Prp_ 0.65, X_Alm_ 0.14, X_Grs_ 0.20 in the core, and X_Prp_ 0.63, X_Alm_ 0.16, X_Grs_ 0.21 in the rim. Its Mg# also shows small variation from 81.2 in the core and 80.2 in the rim.

Amphibole compositions vary from tremolite in Mag-Chl-peridotite and Chr-Chl-peridotite, to tremolite-hornblende in Chr-peridotite and some Chl-Chr-peridotite, to hornblende compositions in Grt-peridotite. In terms of aluminum and titanium content (Fig. 5f), tremolite has the lowest contents (0.01–2.5 wt.% Al_2_O_3_; 0.01–0.2 wt.% TiO_2_), tremolite-hornblende shows intermediate compositions (2.5–8 wt.% Al_2_O_3_; 0.05–0.3 wt.% TiO_2_), and hornblende shows the highest concentrations (9–17 wt.% Al_2_O_3_; 0.25–0.62 wt.% TiO_2_). More rarely, Mg-anthophyllite to anthophyllite is found in Hem-Mag-Chl-peridotite and few Mag- and Chr-Chl-peridotites, measured next to orthopyroxene porphyroblast rims, or forming individual acicular grains. These show MgO between 28.70 and 37.68 wt.%, FeO_tot_ between 3.17 and 7.76 wt.%, SiO_2_ between 55.61 and 59.04 wt.%, and CaO < 0.64 wt.%.

### Magnetite-chromite-spinel mineral series

**Fig. 6:**
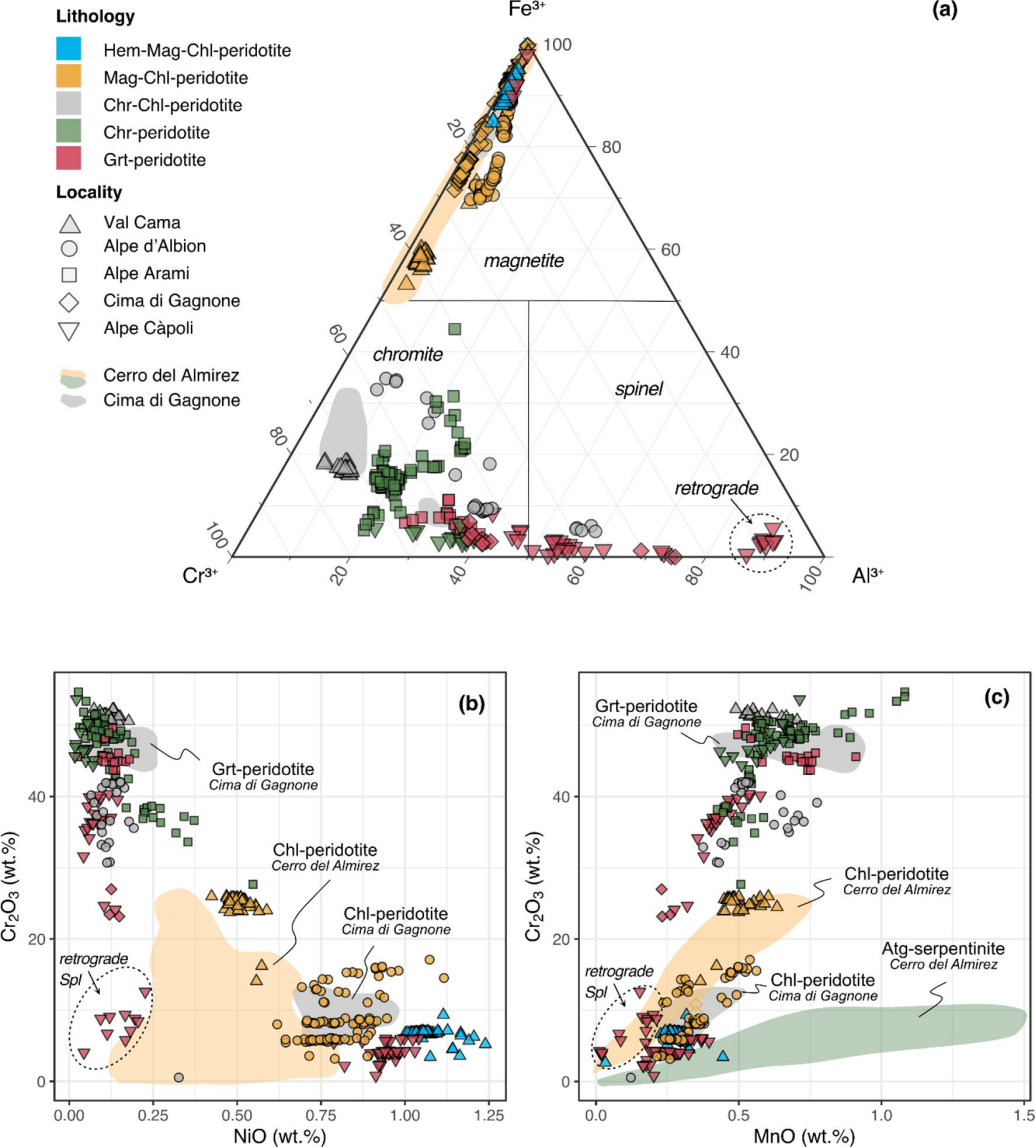
Major element compositions of magnetite, chromite, and spinel for the different lithologies investigated here. Shaded fields represent literature data of Cerro del Almirez (Vieira Duarte et al. 2021) and Cima di Gagnone (Pfiffner 1999). **a** Ternary diagram of magnetite (Fe^3+^), chromite (Cr^3+^), and spinel (Al^3+^) endmembers of spinel group minerals showing the chemical variability of oxides observed in the different lithologies and localities. **b** Variation within Cr_2_O_3_ vs NiO plot, emphasizing NiO enrichments in hematite- and magnetite-enriched oxides relative to chromite-enriched oxides. **c** Variation within Cr_2_O_3_ vs MnO plot, showing the MnO depletion in all investigated samples along with the trends in Almirez Chl-peridotites (Vieira Duarte et al. 2021), which suggest equilibrium of oxides with silicate minerals

The ternary diagram Fe^3+^-Cr^3+^-Al^3+^ (Fig. 6a) shows the classification of the spinel group oxides grouped by sub-lithology (symbols colors) and sampling locality (symbol shapes). Magnetite compositions from Cerro del Almirez (Vieira Duarte et al 2021) and magnetite and chromite compositions from Cima di Gagnone (Pfiffner 1999) are plotted for comparison. Irrespective of locality, which record variable peak pressure and temperature conditions (Fig. 1), an entire range in compositions is observed across the studied samples. Magnetite compositions are characteristic of Hem-Mag-Chl-peridotite and Mag-Chl-peridotite but occur also in one Grt-peridotite. Chromite compositions are found in Chr-Chl-peridotite, Chr-peridotite (Fe-chromite to Al-chromite), and Grt-peridotite (Al-chromite). Finally, Al-spinel compositions are found in interstitial grains or as retrograde associations in symplectites in the Grt-peridotite and exsolutions in magnetite in Mag-Chl-peridotite (not shown in Fig. 6a). Major and minor element compositions of the oxide phases are now explored in terms of their occurrence in the different sub-lithologies (representative compositions are listed in Table 2).

**Table 2:** Representative analyses of hematite, magnetite, chromite, and spinel grains obtained by EPMA in the different metamorphic lithotypes and in different textural positions: inclusions in peak silicates (Incl.) and anhedral interstitial grains (Interst.)

Lithology	Hem-Mag-Chl-per		Mag-Chl-peridotite						Chr-Chl-peridotite		Chr-peridotite		Grt-peridotite		
Sample	PkCa06		Alb18-14	Alb18-09	CdG19-37		PkCa08		Alb18-03		AR18-11	Cap18-01	Cap18-03	AR18-10	CdG19-32
Oxide type	Hem	Mag	Mag	Mag	Cr-Mag	Mag	Cr-Mag	Cr-Mag	Al-Chr	Cr-Spl	Chr	Al-Chr	Mag	Al-Chr	Al-Chr
Text. Position	Incl	Incl	Interst	Incl	Inters	Incl	Interst	Incl	Incl	Interst	Inters	Inters	Inters	Incl	Incl
FeO_tot_	84.41	80.19	79.17	72.53	71.09	91.03	71.37	64.40	27.41	21.90	34.59	22.41	84.94	25.96	22.44
Al_2_O_3_	0.20	0.67	1.91	2.85	3.38	< 0.01	0.81	1.19	20.06	32.45	10.34	20.21	0.46	14.32	21.70
Cr_2_O_3_	2.77	6.65	8.67	11.05	14.35	0.13	17.78	25.98	42.04	32.90	48.09	46.71	4.20	48.71	44.52
TiO_2_	1.93	0.05	0.33	0.39	0.40	0.09	0.37	1.74	0.56	0.13	0.10	0.23	0.18	0.45	0.46
MgO	0.14	5.28	3.19	4.18	3.74	0.75	2.78	1.89	9.37	12.51	5.92	9.25	2.23	8.55	9.58
MnO	0.04	0.23	0.37	0.24	0.31	0.08	0.36	0.60	0.51	0.38	0.61	0.57	0.23	0.50	0.43
NiO	0.04	1.01	0.87	0.76	0.75	0.58	0.67	0.47	0.15	0.11	0.13	0.06	0.99	0.12	0.06
ZnO	< 0.01	0.04	< 0.01	0.06	0.07	0.01	0.11	0.34	0.22	0.27	0.39	0.60	< 0.01	0.49	0.12
Total	89.55	94.12	94.52	92.06	94.10	92.67	94.25	96.62	100.32	100.68	100.17	100.05	93.23	99.09	99.32
*A.p.f.u*															
Fe^3+^	1.86	1.78	1.65	1.53	1.41	1.99	1.42	1.09	0.18	0.10	0.30	0.06	1.84	0.15	0.05
Fe^2+^	0.03	0.68	0.80	0.75	0.77	0.94	0.82	0.90	0.55	0.44	0.67	0.54	0.84	0.56	0.54
Al	0.01	0.03	0.08	0.13	0.15	0.00	0.04	0.05	0.75	1.13	0.41	0.76	0.02	0.56	0.81
Cr	0.06	0.19	0.25	0.33	0.42	0.00	0.53	0.76	1.05	0.77	1.28	1.17	0.13	1.27	1.12
Ti	0.04	0.00	0.01	0.01	0.01	0.00	0.01	0.05	0.01	0.00	0.00	0.01	0.01	0.01	0.01
Mg	0.01	0.29	0.18	0.23	0.21	0.04	0.16	0.10	0.44	0.55	0.30	0.44	0.13	0.42	0.45
Mn	0.00	0.01	0.01	0.01	0.01	0.00	0.01	0.02	0.01	0.01	0.02	0.02	0.01	0.01	0.01
Ni	0.00	0.03	0.03	0.02	0.02	0.02	0.02	0.01	0.00	0.00	0.00	0.00	0.03	0.00	0.00
Zn	0.00	0.00	0.00	0.00	0.00	0.00	0.00	0.01	0.01	0.01	0.01	0.01	0.00	0.01	0.00
Tot	2.00	3.00	3.00	3.00	3.00	3.00	3.00	3.00	3.00	3.00	3.00	3.00	3.00	3.00	3.00
Mag	–	0.89	0.83	0.77	0.71	1.00	0.72	0.57	0.09	0.05	0.15	0.03	0.93	0.08	0.03
Chr	–	0.10	0.13	0.17	0.21	0.00	0.27	0.40	0.53	0.38	0.64	0.59	0.06	0.64	0.56
Spl	–	0.01	0.04	0.06	0.07	0.00	0.02	0.03	0.38	0.56	0.21	0.38	0.01	0.28	0.41

Magnetite component measured as Fe^3+^/(Fe^3+^ + Cr + Al), chromite component as Cr/(Fe^3+^ + Cr + Al), and spinel component as Al/(Fe^3+^ + Cr + Al)

Magnetite compositions in Mag-Chl-peridotite range from nearly pure magnetite (4.57 wt.% Cr_2_O_3_, 84.71 wt.% FeO_tot_, and XFe^3+^ of 0.92, where XFe^3+^  = molar Fe^3+^/[Fe^3+^  + Cr^3+^  + Al^3+^]) to Cr-magnetite (25.39 Cr_2_O_3_ wt.%, 64.54 wt.% FeO_tot_, and XFe^3+^ of 0.58), with NiO contents between ~ 0.46 and 1.00 wt.% (Fig. 6b, except for two outliers at higher NiO). Their MgO contents range from 1.82 to 4.57 wt.% (Table 2), MnO from 0.16 to 0.71 wt.% (Fig. 6c), and Al_2_O_3_ from 0.53 to 3.26 wt.%. Texturally, inclusion-rich cores and inclusion-free rims of large subhedral grains are homogeneous in composition, as shown in the EPMA chemical maps acquired for sample Alb18-11 (Fig. 6a). However, chemical differences can sometimes be observed between magnetite inclusions in peak silicates and interstitial grains within the same sample, showing, as, for example, in sample CdG19-37, Cr_2_O_3_ contents of 7 wt.% to ca. 20 wt.%, respectively (Fig. 6b). Magnetite compositions from this study are similar in Cr, Mn, and Zn, but higher in Ni and Mg relative to magnetite from Cerro del Almirez (Vieira Duarte et al 2021).

Chromite in the Chr-Chl-peridotite and Chr-peridotite has moderate to high Cr_2_O_3_ contents (33.64–51.80 wt.%) and correspondingly moderate to low Al_2_O_3_ (19.48–3.93 wt.%), FeO_tot_ (45.64–23.41 wt.%), and XFe^3+^ (XFe^3+^  < 0.50). It shows also low NiO (0.04–0.32 wt.%), MnO (0.45–0.73 wt.%), and moderate MgO (3.95–9.01 wt.%) and ZnO (0.22–0.59 wt.%). Chromium-spinel is also observed in one sample (Alb18-03), being registered in interstitial grains together with Al-chromite and olivine (Table 2).

Observed oxides in Grt-peridotites are chromite/Cr-spinel (Fig. 6a) and ilmenite. Sample Cap18-03 contains magnetite and ilmeno-hematite. Interstitial chromite and inclusions of chromite in garnet show high Cr_2_O_3_ (45.08–48.88 wt.%) and moderate Al_2_O_3_ (17.74–27.13 wt.%) and MgO (7.16–8.68 wt.%) contents. Their Ni, Mn, and Ti contents are homogeneous, at ca. 0.12 wt.% NiO, and 0.50–0.72 wt.% MnO. Inclusions and interstitial chromite show overlapping Cr and Ni contents, but slightly higher Fe, Mn, Zn, and Ti with lower Al, and Fe in interstitial grains (see Supplementary Table S9). Two Grt-peridotites (Mg160 96-1 and CP16-10) show individual grains with Cr-spinel compositions, with 12.61–40.36 wt.% Cr_2_O_3_, 24.21–56.67 wt.% Al_2_O_3_, and 11.48–24.39 wt.% FeO_tot_. Magnetite in Grt-peridotite Cap18-03 is nearly pure at 84.85 wt.% FeO_tot_, 4.43 wt.% Cr_2_O_3_, 0.46 wt.% Al_2_O_3_, and 2.44 wt.% MgO.

Green spinel in symplectites forming around garnet during retrogression shows Al-rich compositions with ca. 55.95–59.16 wt.% Al_2_O_3_, 18.14–23.86 wt.% MgO, 7.44–13.37 wt.% FeO_tot_, and 7.59–12.02 wt.% Cr_2_O_3_.

### Ilmenite-hematite solid solution minerals

Minerals from the ilmenite-hematite group have been identified in seven samples from all the metamorphic lithotypes, with exception of Chr-Chl-peridotite and Chr-peridotite (Table 1). Pure hematite (Hem_96-99_Ilm_4-1_) occurs interstitially and as inclusion in olivine, orthopyroxene, and chlorite in the Hem-Mag-Chl-peridotite (sample PkCa-06; Table 2). It shows elevated Cr (2.82 wt.% Cr_2_O_3_) and measurable Al (0.17 wt.% Al_2_O_3_) contents, and very low Mg, Mn, and Ni (< 0.01 wt.%).

Crystals dominated by the ilmenite component (Hem_<11_Ilm_>89_) are the most common across the different metamorphic lithotypes, reaching pure ilmenite compositions in the Grt-peridotite. Pure ilmenite (< 2.5% hematite component) shows the lowest Cr (< 0.5 wt.% Cr_2_O_3_) and Al (< 0.1 wt.% Al_2_O_3_) and variable MgO, MnO, and NiO contents of 4.81–9.34, 0.05–0.50, and 0.05–0.21 wt.%, respectively, which are generally lower in interstitial grains compared to inclusions. In Mag-Chl-peridotite ilmenite compositions show variable Cr_2_O_3_ (0.19–0.99 wt.%), Al_2_O_3_ (0–0.15 wt.%), and NiO (0.07–0.19 wt.%), but uniform MgO (5.76 wt.%) and MnO (0.71wt.%) contents.

Ilmenite-hematite solid solution crystals displaying retrograde exsolution textures as reported for Almirez (Vieira Duarte et al 2021) are found in one Mag-Chl-peridotite (sample CdG19-50) showing Hem_66-74_Ilm_34-26_ and Hem_6-7_Ilm_94-93_ compositions and in one Grt-peridotite (samples Cap18-03) showing Hem_75-81_Ilm_25-19_ and Hem_7_Ilm_93_ compositions (Fig. 3h). Reintegration of the pre-exsolution compositions, considering 10 and 18 vol.% of ilmenite components retrieved by image analysis, gives Hem_55-67_Ilm_45-33_ for Mag-Chl-peridotite, and Hem_63-74_Ilm_37-26_ for Grt-peridotite, respectively.

### Sulfide minerals

Pentlandite is the dominant sulfide, while pyrrhotite is rarely observed. Sulfide measurement data for Chr-Chl-peridotites and some Grt-peridotites are shown in Table 3. Recall that no sulfide was observed in the Hem-Mag-Chl-peridotite and Mag-Chl-peridotite (Table 1).

**Table 3:** Representative analyses of sulfide minerals obtained by EPMA in the different metamorphic lithotypes

Lithology	Chr-Chl-per	Chr-peridotite					Grt-peridotite	
Sample	PkCa01	AR18-11	AR18-07		Cap18-01		AR18-10	
Sulfide type	Pn	Pn	Pn	Hzl	Pn	Hzl	Pn	Po
*Wt.%*								
S	33.41	32.90	33.38	26.78	33.47	27.29	33.37	36.52
Fe	37.98	31.16	32.02	1.82	32.80	1.16	42.52	62.17
Pb	0.37	0.41	0.46	0.30	0.40	0.43	0.40	0.58
Cu	0.38	< 0.01	0.04	< 0.01	0.07	< 0.01	0.58	0.22
Ni	27.23	35.37	33.02	72.17	32.33	72.42	23.18	0.89
Co	0.48	0.31	0.46	< 0.01	0.89	0.02	0.37	0.07
Zn	0.01	< 0.01	0.03	0.04	0.03	0.03	< 0.01	< 0.01
Total	99.86	100.15	99.41	101.11	100.00	101.35	100.42	100.45
*A.p.f.u*								
S	8.00	8.00	8.00	2.00	8.00	2.00	8.00	1.00
Fe	5.22	4.70	4.32	2.94	4.22	2.90	3.04	0.01
Pb	0.01	0.00	0.00	0.00	0.00	0.00	0.00	0.00
Cu	0.05	0.02	0.02	0.00	0.01	0.00	0.01	0.00
Ni	3.56	4.35	4.41	0.08	4.50	0.05	5.85	0.98
Co	0.06	0.04	0.06	0.00	0.12	0.00	0.05	0.00
Zn	0.00	0.00	0.01	0.00	0.01	0.00	0.07	0.00
Total	16.91	17.10	16.81	5.03	16.87	4.96	17.02	2.00
Ni/Fe	0.68	0.93	1.02		1.07		1.93	

Pentlandite is the principal sulfide found in Chr-Chl-peridotite, Chr-peridotite, and Grt-peridotite. Pentlandite in Grt-peridotite shows distinct compositional ranges with high Fe contents (> 37 wt.%) relative to corresponding Ni contents (< 29 wt.%; Table 3), while Chr-Chl-peridotite and Chr-peridotite display a wider range in Ni (25–36 wt.%) and Fe (30–40 wt.%) contents. However, all samples show similar Cu (up to 2.85 wt.%) and Co (0.25–0.96 wt.%). Pyrrhotite was also found in Grt-peridotites, where it shows Fe between 61.51 and 64.11 wt.%, S between 35.85 and 38.12 wt.%, and variably low Ni (< 0.89 wt.%), Co (< 0.08 wt.%), and Cu (< 0.22 wt.%).

Rare heazlewoodite was identified by measurement in one Chr-peridotite and one Grt-peridotite (samples AR18-07 and Cap18-01), in grains previously identified during microscopy as prograde pentlandite. They show similar compositions with 72.42 wt.% Ni, 27.29 wt.% S, 1.16 wt.% Fe, 0.43 wt.% Pb, and very low Cu, Co, and Zn.

### LA-ICP-MS measurement data of oxides

#### Magnetite-chromite-spinel group

A large range in lithophile, siderophile, and chalcophile elements was detected in oxides of the magnetite-chromite-spinel group by LA-ICP-MS (Supplementary Figure S3). Enrichments relative to primitive mantle (PM, Palme and O’Neill 2014) are observed in Fe, Cr, Al, Ni, Mn, Zn (up to 280 PM), Co (up to 6 PM), V (up to 50 PM), Ga (up to 24 PM), Ti, Sn (up to 150 PM), Mo (up to 15 PM), As (up to 50 PM), Pb (up to 290 PM), W (up to 52 PM), and U (up to 32 PM). Major differences are observed between ranges in the different metamorphic lithotypes. Despite the ranges observed for major elements Al, Cr, Fe, and Ni, a general increase occurs variably from Hem-Mag-Chl-peridotite to Grt-peridotite in V (800–2300 µg g^−1^), Ga (10–87 µg g^−1^), Zr (0.06–0.32 µg g^−1^), and Nb (0.044–0.28 µg g^−1^), along with a decrease in Cu (10–0.36 µg g^−1^) and Sc (20–2 µg g^−1^).

### Ilmenite-hematite solid solution minerals

Minor to trace element composition of the ilmenite-hematite solid solution minerals were measured by LA-ICP-MS in three samples, and data normalized to Primitive Mantle (Palme and O’Neill 2014) are displayed in Supplementary Figure S3.

Pure hematite from Hem-Mag-Chl-peridotite (sample PkCa-06) shows the highest enrichments in Sc (97 µg g^−1^), and the lowest in Co (4 µg g^−1^), Zn (1 µg g^−1^), Mn (21 µg g^−1^), and Ni (64 µg g^−1^) relative to all hematite-ilmenite, pure ilmenite, and magnetite-chromite-spinel minerals. Pre-exsolution ilmeno-hematite compositions measured in the Grt-peridotite sample Cap18-03 were done using a 20 µm beam size. This covers a volume that is much larger than the size of the exsolution lamellae and thus is representative of the bulk composition of the grains. These compositions are similar to ilmeno-hematite reported for Chl-peridotite from Cerro del Almirez (Vieira Duarte et al 2021), except for lower Ti contents. The composition of bulk ilmeno-hematite is prominently different to those of pure hematite found in Hem-Mag-Chl-peridotite, showing higher values for a series of elements, such as, Nb, Sn, Ti, V, Sc, Co, Zn, Mn, and Ni, and lower values for W and Zr (Supplementary Figure S3).

Peak ilmenite shows the highest enrichments in W (up to 7 µg g^−1^), Nb (up to 340 µg g^−1^), Zr (up to 160 µg g^−1^), Y (up to 0.34 µg g^−1^), and Mn (up to 30,600 µg g^−1^), and the lowest Ga (0.031–0.28 µg g^−1^), Al (0.01–51 µg g^−1^), and Cr (350–3500 µg g^−1^) in comparison to hematite and magnetite-chromite-spinel minerals. Ilmenite compositions overall agree with the ranges reported for Chl-peridotites from Cerro del Almirez (Vieira Duarte et al 2021), only showing higher values for W, Zn, Mn, and Ni, and lower Sn, Ga, Y, V, Sc, Co, Zn, Al, and Cr (see Supplementary Figure S3).

## Discussion

### Compositional variability of metaperidotites

The metaperidotite bodies of former oceanic lithosphere investigated here record a complex history, involving processes of melt depletion, metasomatism upon ocean floor hydration, prograde modifications during subduction, and variable retrogression during exhumation. Here we summarize the most relevant information. Bulk rock data (Supplementary Table S2) cover the range from lherzolitic to harzburgitic and dunitic compositions, which plot along the melting residue trend from primitive mantle compositions (Niu 2004; Palme and O’Neill 2014), as shown in MgO/SiO_2_ vs. Al_2_O_3_/SiO_2_ and Al_2_O_3_ vs. CaO plots (Fig. 4a, c). Low MgO/SiO_2_ values for the given Al_2_O_3_/SiO_2_ ratios overlap with the oceanic peridotite array (Niu 2004; Kodolanyi et al. 2012), which can be explained either (i) by addition of SiO_2_ during ocean floor hydration or (ii) by MgO loss during low temperature seafloor weathering. The first process seems to be predominant as no correlation is apparent between the bulk rock Mg# and the deviation from the melt residual trend (Fig. 4a) as has been demonstrated for Almirez metaperidotites (Pettke and Bretscher 2022), consistent with the findings of Malvoisin (2015). A few samples display higher MgO/SiO_2_ for its corresponding Al_2_O_3_/SiO_2_ (notably Alb18-10), which is here interpreted to represent replacive dunite formation rather than residual dunites. This is indicated by olivine modes of up to 87 vol.% and Mg# of 87.6–88.9. This Mg# is much lower than those characteristic for residual dunite, i.e., displaying a relative enrichment in FeO (Fig. 4b; compare, e.g., Su et al 2016). A plot of CaO vs. Al_2_O_3_ (Fig. 4c) reveals that some samples align along the melt depletion trend while others display a CaO depletion typical for Ca loss upon oceanic serpentinization (Coleman and Keith 1971).

These magmatic and serpentinization-related variabilities demonstrate that mantle rocks that eventually get subducted are compositionally heterogeneous materials, which can influence the changes in composition and modes of oxides, sulfides, and silicates during prograde metamorphic reactions. To constrain the redox conditions of serpentinite dehydration reactions during subduction, it is crucial to address these pre-subduction variability factors using combined proxies (see below).

### Olivine Mg# and oxide mode as combined proxies for metaperidotite redox budget

**Fig. 7:**
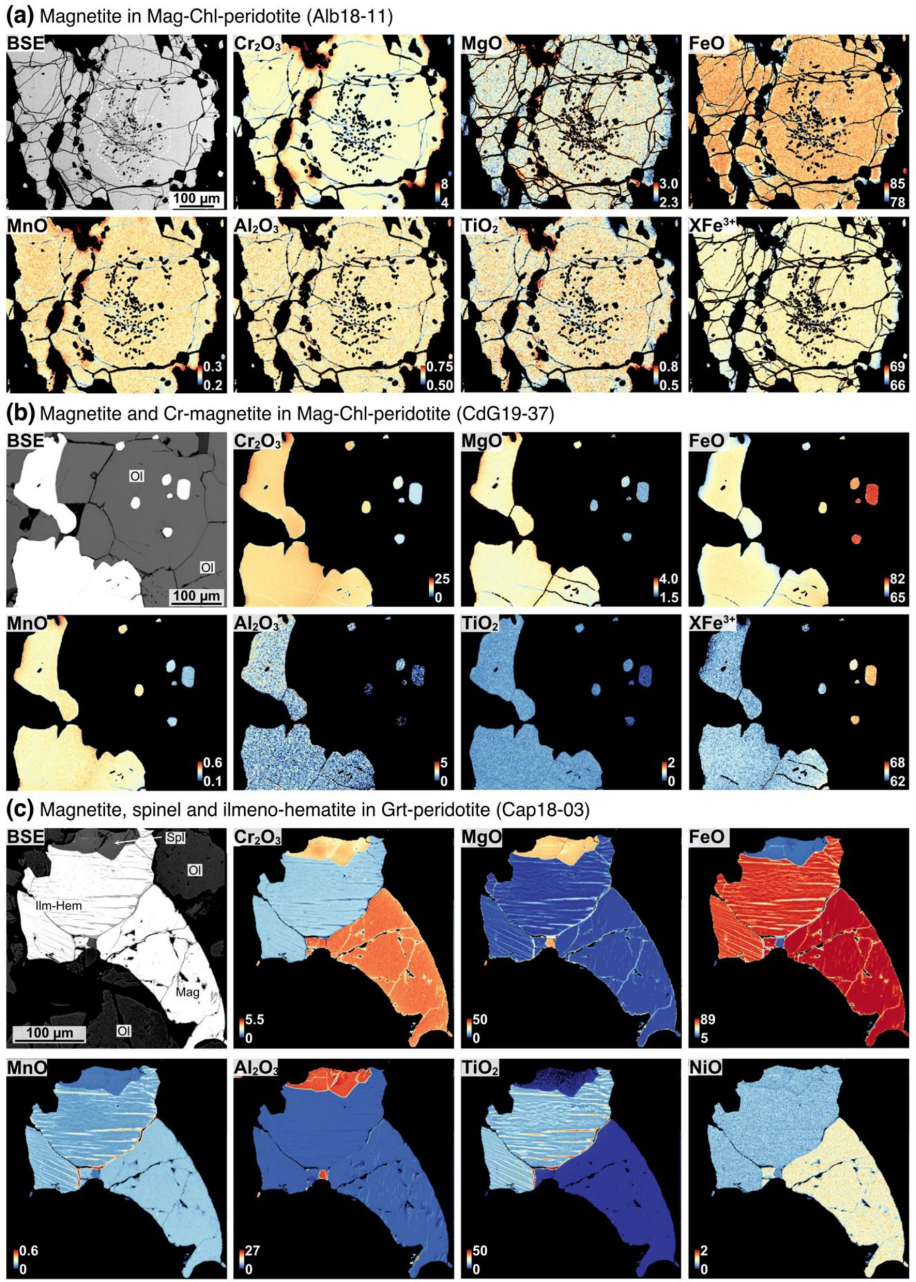
Compositional X-Ray EPMA maps of oxide grains, showing concentrations in oxide wt.%. **a** Subhedral magnetite in Mag-Chl-peridotite of Alpe Albion, showing uniform composition for inclusion-rich core and inclusion-free rim. **b** Inclusion and subhedral Cr-magnetite in Mag-Chl-peridotite of Cima di Gagnone. **c** Intergrowths of magnetite, ilmeno-hematite, and retrograde spinel in former Grt-peridotite of Alpe Capoli

**Fig. 8:**
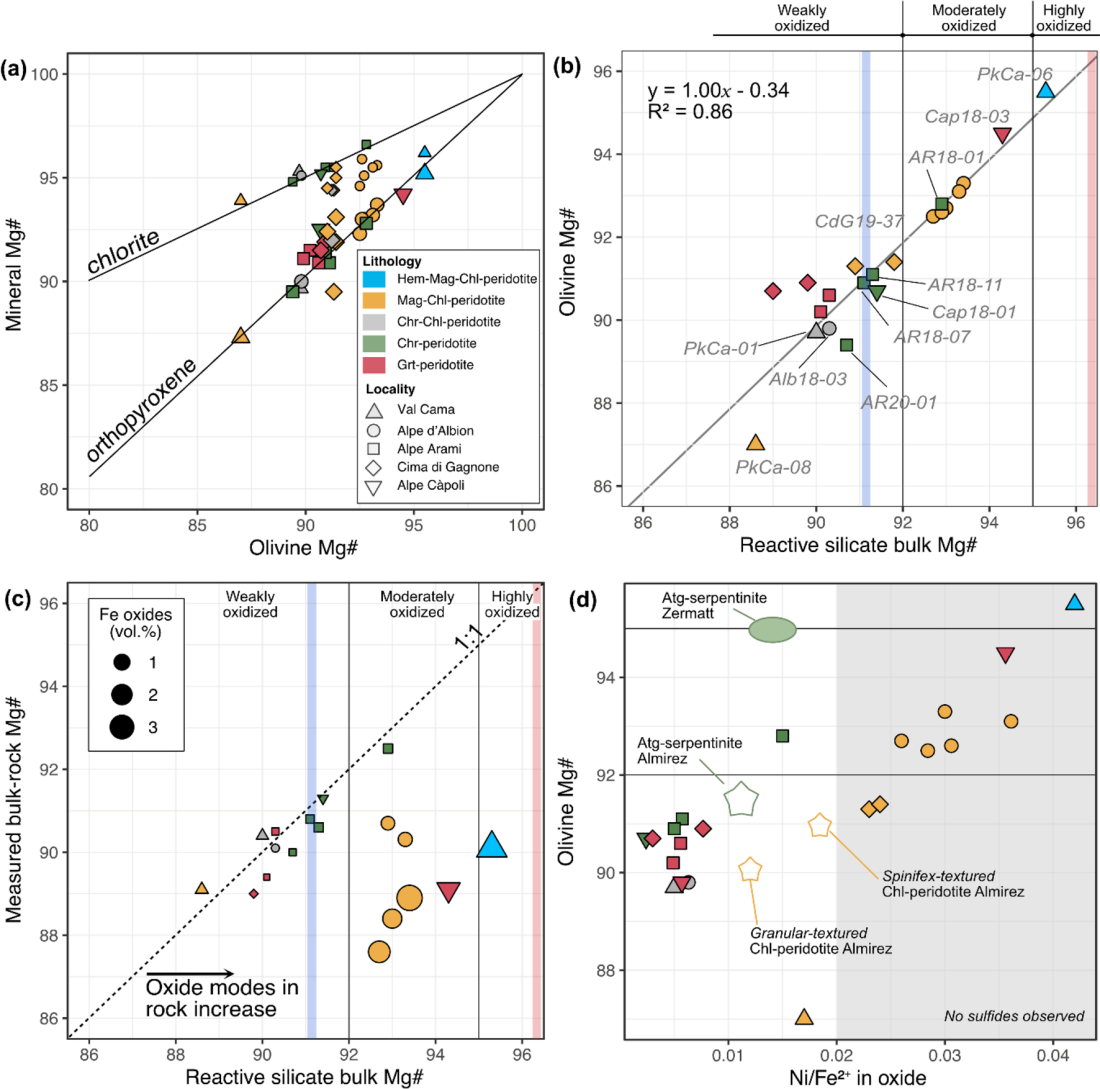
Compositional relationships. **a** Olivine Mg# vs. mineral Mg# as labeled on the correlation lines given by Trommsdorff and Evans (1972). **b** Average olivine Mg# vs. reactive silicate bulk Mg# for each metaperidotite. Colored bars represent the reactive silicate bulk composition of the oxidized (red) and reduced (blue) case modeled in Evans and Frost (2021). **c** Measured bulk rock Mg# vs. reactive silicate bulk Mg#. The extent of horizontal deviation from the 1:1 line (black arrow) of a given sample correlates with the fraction of iron bound in oxide minerals (as emphasized by the size of the symbols). **d** Averaged olivine Mg# and Ni/Fe^2+^ ratios in coexisting oxides, obtained by stoichiometry. Averaged sulfide and magnetite-bearing Atg-serpentinite and Chl-peridotite compositions from Cerro del Almirez (Bretscher et al. 2018; Vieira Duarte et al. 2021) and Atg-serpentinite from Zermatt (Kempf et al. 2020) are also plotted for comparison. Gray area emphasizes the conditions at which no sulfide minerals were observed in the metaperidotites investigated here

Silicate minerals in the diverse metaperidotite lithologies display some remarkable major element systematics (Fig. 7). Averaged olivine and orthopyroxene Mg# align along near the 1:1 line defined for metaperidotites (Fig. 8a; Kempf et al 2022), demonstrating equilibrium between these two silicate minerals in all samples. Highest Mg# in olivine and orthopyroxene are found in Hem-Mag-Chl-peridotite sample PkCa-06 (average Mg# of 95.5 and 95.2 for olivine and orthopyroxene, respectively) and one Grt-peridotite (sample Cap18-03; average Mg# of 94.5 and 94.2 for olivine and orthopyroxene, respectively). In the Mag-Chl-peridotite, moderate to high Mg# of 91.3–93.3 in olivine and of 89.2–93.7 in orthopyroxene are observed (with the exception of 87.0 and 87.3, respectively, for olivine and orthopyroxene of sample PkCa-08). These values overlap and extend to more magnesian values when compared with “mantle-like” Mg# observed in olivine (89.4–91.1) and orthopyroxene (89.5–92.5) in Chr-Chl-peridotite, in the majority of Grt-peridotite, and Chr-peridotite, with the exception of Chr-peridotite AR18-01 representing a residual dunite with high olivine and orthopyroxene Mg# of ~ 93 and the replacive dunite PkCa-08 at ~ 89.1. However, the relations between olivine and chlorite Mg# are not so well defined, showing lower Mg# in chlorite relative to the metaperidotites trend of Trommsdorff and Evans (1972), especially in Hem-Mag-Chl-peridotite and some Mag-Chl-peridotite (Fig. 8a). Nevertheless, we consider all inter-mineral Mg# systematics to be representative because all rocks display well-equilibrated textures, often showing straight and typical 120° ternary grain boundaries.

Measured olivine Mg# correlates with reactive silicate bulk Mg# (Fig. 8b) along the 1:1 line. The reactive silicate bulk Mg# quantifies the influence of bulk Fe and Mg chemistry based on the silicate minerals that participate in the antigorite and chlorite dehydration reactions. It is calculated, as developed in Bretscher et al (2018), by considering the measured silicate mineral Mg# of olivine, orthopyroxene, and chlorite proportionally to their modes, i.e., without the oxide minerals (see also Tracy 1982; Lanari and Engi 2017). A clear correlation disappears when measured bulk rock Mg# is plotted against its corresponding reactive silicate bulk Mg# (Fig. 8c). The extent of deviation from the 1:1 line is a function of oxide modes shown in the inset of Fig. 8c: the higher the oxide modes, the farther the data points are shifted to the right of the 1:1 line toward higher reactive silicate bulk Mg#. This readily illustrates the effect of magnetite formation upon oceanic serpentinization in that Fe is transferred from the silicate fraction of the precursor peridotite into newly formed oxides (i.e., magnetite and hematite) in the serpentinite. The deviation from the 1:1 line in Fig. 8c thus gives an indication on how much Fe is fixed in oxides, offering a first-order proxy on how strongly the metaperidotites are oxidized relative to precursor peridotite. Measured bulk rock Mg# are within the magmatic variability discussed before, being well constrained between ~ 89 and 90, except for the replacive dunites at ~ 87–89 and the residual dunite at ~ 93, respectively.

It has been documented that the reactive silicate bulk Mg# as well as the Mg# of olivine and orthopyroxene can serve as proxy for oxide modes equilibrated in the rock (Bretscher et al 2018; Vieira Duarte et al 2021). This work validates the previous results over a wider range of localities and peak P–T conditions. The highest reactive silicate bulk Mg# and oxide modes are found in Hem-Mag-Chl-peridotite PkCa-06 (95.3 and ~ 2.70 vol.% Mag + Hem) and Grt-peridotite Cap18-03 (94.3 and ~ 0.80 vol.% Mag ± Ilm-Hem), followed by Mag-Chl-peridotite (reactive silicate bulk Mg# 91.8–93.5, and 0.48–3.58 vol.% Mag). In contrast, lower reactive silicate bulk Mg# and oxide modes are found in Chr-Chl-peridotite (89.7–91.3, and 0.10–0.38 vol.% Chr) and Grt-peridotite (89.4–90.5, and 0.06–0.11 wt.% Chr).

**Fig. 9:**
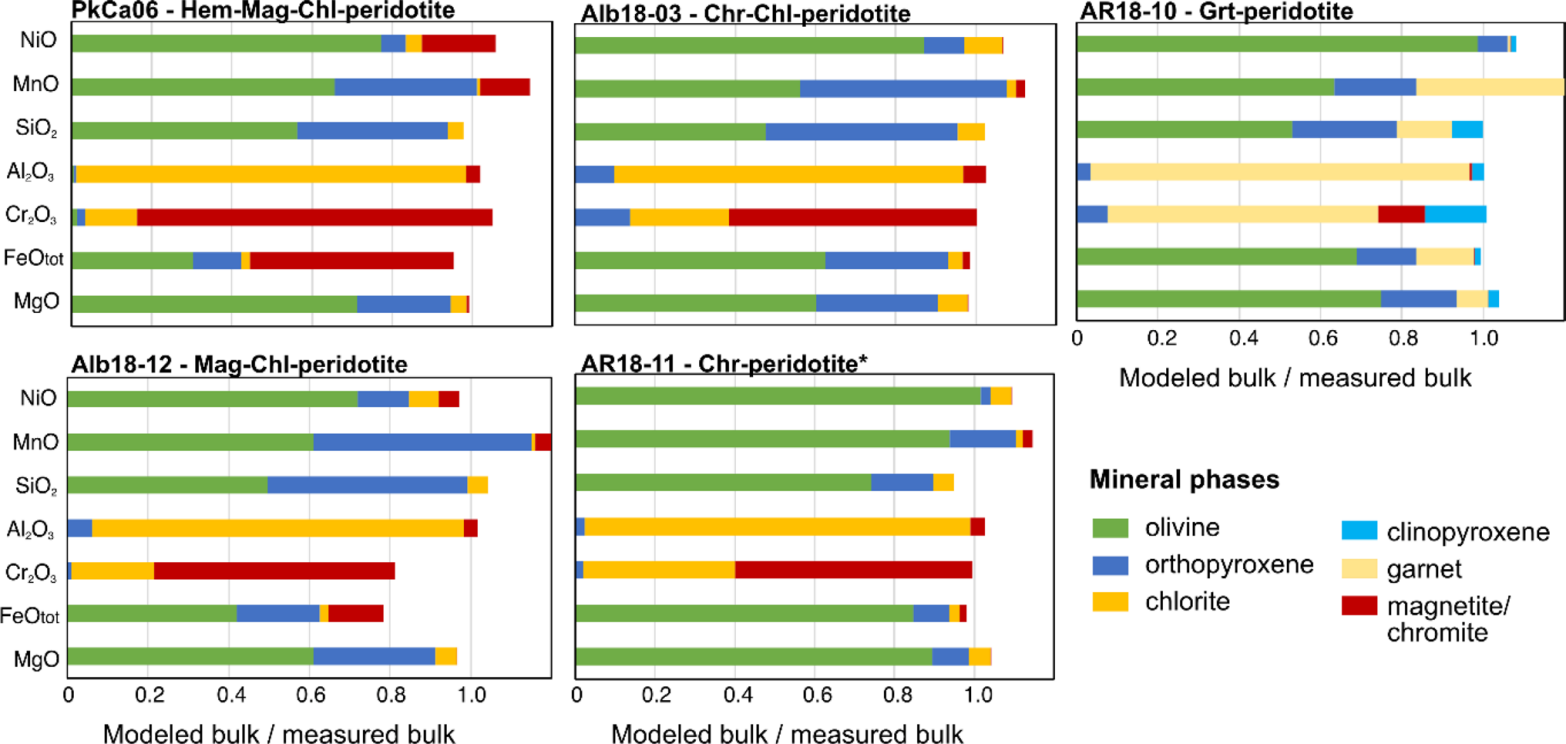
Element distribution diagrams between peak mineral assemblages for major element oxides represented in selected samples. Data were plotted for Hem-Mag-Chl-peridotite (PkCa06), Mag-Chl-peridotite (Alb18-02), Chr-Chl-peridotite (Alb18-03), Chr-peridotite (AR18-11), and Grt-peridotite (AR18-10). Modeled bulk compositions represent the sum of weighted element oxide compositions of all peak metamorphic minerals (amphibole excluded here); and measured bulk corresponds to the measured bulk rock data obtained in this study. *Recall that chlorite in Chr-peridotite is retrograde (Table 1). Amphibole is not displayed as it does not represent a significant host for the elements and samples on a bulk rock scale

Samples plotting along the 1:1 line in Fig. 8c can be interpreted in two ways. In the first, they had (very) low initial oxide contents resulting from lower extents of oxidation upon oceanic hydration. In the second, their reactive silicate bulk chemistry was adjusted during prograde metamorphism in the Chl-peridotite stability field as a consequence of progressively decreasing oxide modes, with potential iron redistribution or even transfer of redox budget to the dehydration fluids. From the silicate mineralogy alone, it is not straightforward to deduce if Chl-peridotites and Grt-peridotites (which plot on the 1:1 line in Fig. 8c) had lower initial oxide contents resulting from lower extents of oxidation upon hydration. To clarify the role of silicate and oxide phases, peak metamorphic element distribution diagrams were calculated (Fig. 9). Oxide phases are most relevant for bulk rock Fe and Cr, being minor hosts of Al, which is nearly fully hosted in chlorite or garnet, and less in orthopyroxene. Magnetite in Mag-Chl-peridotites can host up to ~ 50% of total bulk rock Fe, and variable amounts of Cr. In turn, chromite in Chr-Chl-peridotites and Grt-peridotite is variably significant for Cr (depending on garnet stability), but it is irrelevant for Fe, which is essentially bound to olivine, orthopyroxene, and/or garnet. Therefore, the ferric iron incorporated into these latter silicate minerals would be important to fully quantify the redox budget.

**Fig. 10:**
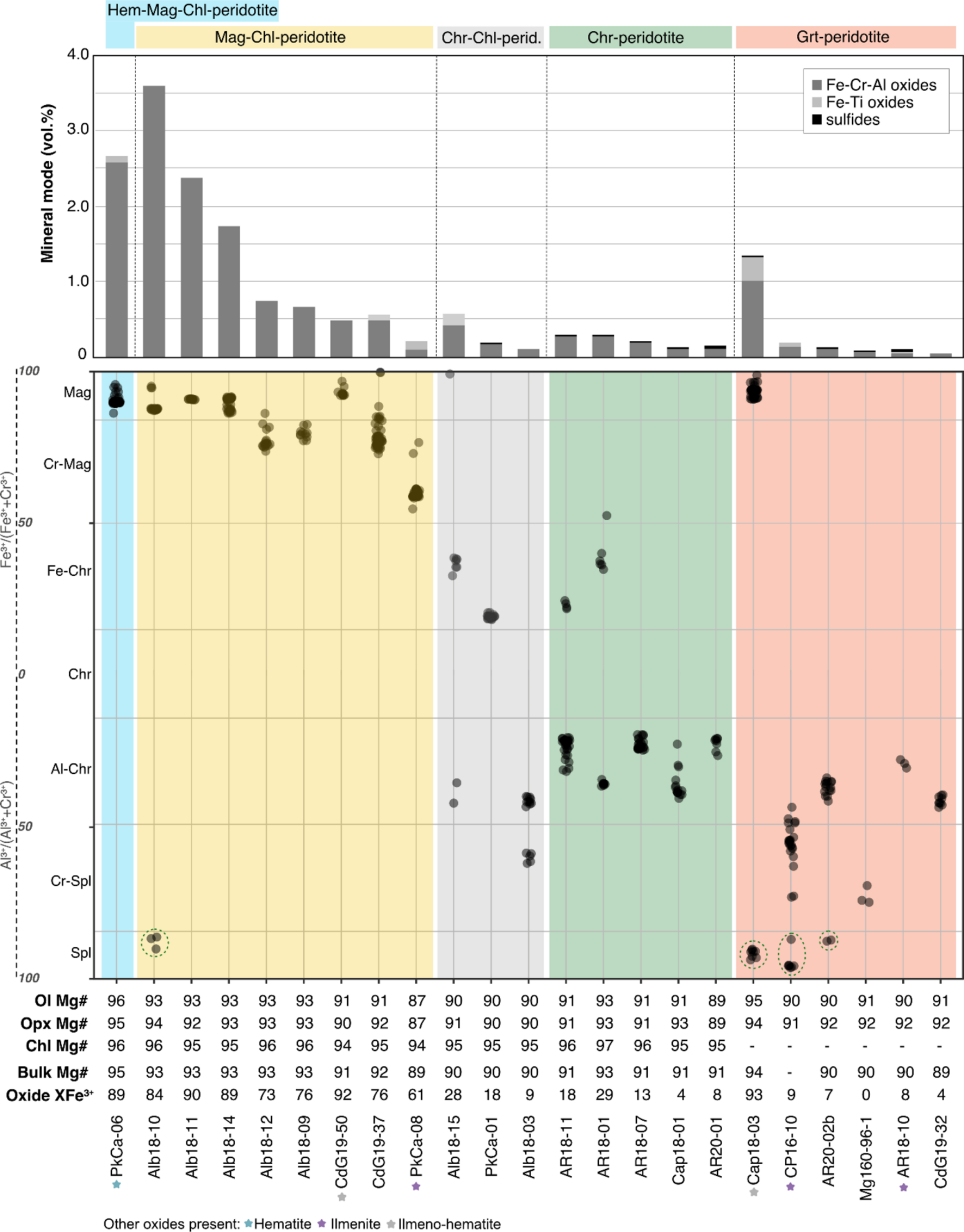
Schematic diagram showing the oxide mineralogy present in the studied samples (Y-axis), sorted by decreasing total oxide mode (in vol.%) for each lithology group, represented by colored bands: light blue for Hem-Mag-Chl-peridotite, yellow for Mag-Chl-peridotite, gray for Chr-Chl-peridotite, green for Chr-peridotites, and red for Grt-peridotite. The Mg# of coexisting olivine, orthopyroxene, and chlorite given as molar Mg/(Mg + Fetot), as well as the reactive silicate bulk rock Mg#, and the spinel XFe^3+^ (XFe^3+^  = molar Fe^3+^/(Fe^3+^  + Cr^3+^  + Al^3+^)100) are also indicated. Colored stars next to the sample label indicate the presence of hematite, ilmenite, and ilmeno-hematite minerals as defined at the bottom of the figure

Figure 10 presents a schematic view of the main oxide chemistry of the magnetite-chromite-spinel group in the different samples (y-axis) classified in the metamorphic lithotypes (colored vertical bands), sorted by decreasing total oxide mode (x-axis), and in relation to the Mg# of coexisting olivine, orthopyroxene, and chlorite. The order of the given metamorphic lithotypes, from Hem-Mag-Chl-peridotite (on the left) to Chr- and Grt-peridotite (on the right), highlights their decreasing reactive silicate bulk Mg# with increasing metamorphic grade. An overall decrease in Fe^3+^ content in oxide with decreasing mode is observed from Hem-Mag-Chl-peridotite and Mag-Chl-peridotite to Chr-Chl-peridotite, and Grt-peridotite. Magnetite-bearing samples show oxide modes up to 3.6 vol.%, while chromite-, spinel-, and garnet-bearing samples show generally lower oxide modes (< 0.5 vol.%), with exception of one Grt-peridotite (Cap18-03).

In summary, the abundance and composition of the oxide phase correlate well with the olivine Mg# (Fig. 10) and the olivine Mg# is in turn a good proxy for the Mg# of the reactive silicate bulk. This information gives a minimum estimation on how much ferric iron is present in the rock. However, to assess a full redox budget, especially of the less oxidized samples, ferric iron measurements in silicates would be required.

### Oxide geochemistry from antigorite-out to beyond chlorite-out reactions

It was demonstrated before that during subduction of weakly oxidized, serpentinized peridotites, magnetite recrystallizes upon antigorite dehydration. New magnetite is formed together with olivine, orthopyroxene, and chlorite as a consequence of the redistribution of Fe^3+^, Cr, Al, and V liberated from reactant antigorite (Vieira Duarte et al 2021). Magnetite is also observed in Chl-peridotites in most of the sampled ultramafic lenses in the Central Alps, except for the samples of Alpe Arami. Texturally, magnetite occurs as inclusions (Fig. 3a–c) in olivine, orthopyroxene, and chlorite, but can also show large polygonal aggregates with the core rich in chlorite inclusions and inclusion-free rim, similar to magnetite textures observed in the Atg-serpentinites of Cerro del Almirez (Vieira Duarte et al 2021). However, despite the textural variability, magnetite from the Central Alps shows well-equilibrated, homogeneous compositions (Fig. 7a, b), with narrow ranges for each sample (Fig. 10), in contrast to the striking chemical zoning documented for polygonal magnetite compositions from Chl-peridotite of Almirez (Vieira Duarte et al 2021). In the central Alps samples, an evolution in magnetite composition can sometimes be recognized between texturally different magnetite types, e.g., Fe-rich magnetite inclusions enclosed in silicates occur together with Cr-enriched magnetite present as anhedral grains in interstitial position (Fig. 7b).

The most conspicuous chemical variation in magnetite is the fraction of chromite component in magnetite, which can be influenced by different factors. These include as follows: (i) the initial oxide mineral modes, which are dependent on the extent of oxidation during oceanic serpentinization; (ii) the variations in Cr concentrations in bulk rock peridotite; and (iii) the possible Cr-Fe^3+^-Al element redistribution between silicate and oxide minerals across metamorphic reactions. The chromite component in magnetite is important because it influences the redox buffer conditions, whereby higher Cr_2_O_3_ contents go along with lower *f*O_2_, as will be developed in the next section.

Moreover, while relative enrichments in magnetite Mg and Al do not show clear relation to their Cr_2_O_3_ (Figs. 4 and 6) nor to bulk rock concentrations, they might monitor progressive chlorite decomposition with increasing pressure and temperature conditions. Accordingly, enrichments such as Mn and Ni can be useful indicators of the stable mineral assemblage during prograde conditions. Manganese enrichments correlate with chromium, following the same trend observed for Chl-peridotites in Cerro del Almirez (Fig. 6c), thus indicating equilibrium with reaction product olivine and orthopyroxene, which preferentially incorporate these elements (Vieira Duarte et al 2021). Nickel enrichments display trends identical to those of Mg and Co, being extremely variable (notably in PkCa-06) and in some samples showing higher Ni contents in magnetite inclusions when compared to interstitial and anhedral grains. Variable Ni and Co contents can be related to sulfide stability in the metaperidotites as discussed below.

### Silicate-oxide relationships across the continuous Chl-out reactions

Remarkable differences are observed between magnetite-bearing and chromite-bearing metaperidotites. Magnetite-bearing metaperidotites generally show higher oxide modes (up to 3.6 vol.%) and higher reactive silicate bulk Mg# (90.9–94.3), while chromite-bearing metaperidotites show lower oxide modes (< 0.4 vol.%) along with lower reactive silicate bulk Mg# (90.0–91.4) (Fig. 10). Moreover, characteristic differences are also observed for Al, Cr, and FeO_tot_ concentrations in coexisting silicate and oxide minerals (Figs. 4 and 5).

Differences in oxide mineral composition between magnetite and chromite, and the Fe distribution between rock-forming minerals, may reflect the prograde metamorphic evolution of the rocks. A progressive increase in Cr_2_O_3_ concentration of magnetite could record progressive consumption of chlorite whose Cr_2_O_3_/FeO_tot_ concentration ratio is of the order of 0.5, i.e., much higher than that of olivine and orthopyroxene (< 0.02; Supplementary Table S9). The following exchange reactions (at constant redox budget) may be relevant:

4 $${\text{Ca}}_{{2}} {\text{Mg}}_{{5}} {\text{Si}}_{{8}} {\text{O}}_{{{22}}} \left( {{\text{OH}}} \right)_{{2}} + {\text{ Mg}}_{{5}} {\text{Al}}_{{2}} {\text{Si}}_{{3}} {\text{O}}_{{{1}0}} \left( {{\text{OH}}} \right)_{{8}} = {\text{Ca}}_{{2}} \left( {{\text{Mg}}_{{4}} {\text{Al}}} \right)\left( {{\text{Si}}_{{7}} {\text{Al}}} \right){\text{O}}_{{{22}}} \left( {{\text{OH}}} \right)_{{2}} + {\text{ 2Mg}}_{{2}} {\text{SiO}}_{{4}} + {\text{ Mg}}_{{2}} {\text{Si}}_{{2}} {\text{O}}_{{6}} + {\text{ 4H}}_{{2}} {\text{O,}}$$

5 $${\text{Mg}}_{{5}} {\text{Al}}_{{2}} {\text{Si}}_{{3}} {\text{O}}_{{{1}0}} \left( {{\text{OH}}} \right)_{{8}} = {\text{2Mg}}_{{2}} {\text{SiO}}_{{4}} + {\text{ MgAl}}_{{2}} {\text{SiO}}_{{6}} + {\text{ 4H}}_{{2}} {\text{O,}}$$

6 $${\text{Mg}}_{{5}} {\text{Fe}}^{{{3} + }} {\text{CrSi}}_{{3}} {\text{O}}_{{{1}0}} \left( {{\text{OH}}} \right)_{{8}} = {\text{MgFe}}^{{{3} + }} {\text{CrO}}_{{4}} + {\text{ Mg}}_{{2}} {\text{Si}}_{{2}} {\text{O}}_{{6}} + {\text{ Mg}}_{{2}} {\text{SiO}}_{{4}} + {\text{ 4H}}_{{2}} {\text{O}}{.}$$

Reaction (4) describes the equilibrium transformation of tremolite + chlorite to Mg-hornblende via a Tschermak exchange (Al_2_Mg_-1_Si_-1_) + forsterite + enstatite + water. Reaction (5) reports chlorite decomposition to forsterite + Al component in enstatite via a Tschermak exchange (Al_2_Mg_-1_Si_-1_) + water. Both reactions thus infer progressive consumption of chlorite, with enrichment of Al in amphibole and orthopyroxene. Because chlorite contains Cr_2_O_3_ that is not taken up in reaction product minerals of Eqs. 4 and 5 (except subordinately for Mg-hornblende beyond chlorite out; Supplementary Tables S6 and S8), the chromium along with some Mg liberated from chlorite is likely transferred to the oxide mineral (Eq. 6). This could either increase oxide modes or could render the oxide mineral progressively enriched in Cr via an exchange reaction of (Cr^3+^-Mg^2+^) for (Fe^3+^-Fe^2+^), while the iron liberated from the oxide might be redistributed into coexisting silicates.

Increasing Al_2_O_3_ with increasing TiO_2_ in amphibole are indicative of increasing temperature (Liao et al 2021) as shown by evolving compositions from tremolite to Mg-hornblende in higher grade rocks (Fig. 5f). Such an evolution with temperature is supported by compositional trends of increasing Al_2_O_3_ from core to rim in orthopyroxene (Al_2_O_3_ is a trace element in orthopyroxene; Supplementary Figure S4) in some Chr-Chl-peridotite, Chr-peridotite, and Grt-peridotite of our study. The Mg concentration of the oxide mineral generally increases along with its Cr concentration (Supplementary Table S9). To the contrast, our data do not allow to test whether the Mg# of olivine and orthopyroxene evolved toward lower values with progressive chlorite decomposition, which would monitor uptake of iron liberated from the oxide mineral upon re-equilibration. This is because olivine and orthopyroxene are unzoned and because their starting Mg# prior to chlorite decomposition (i.e., that formed after antigorite dehydration) is not known for a given sample. Moreover, significant water is liberated by continuous chlorite decomposition that keeps the rocks wet until complete chlorite consumption, thus catalyzing constant equilibration and recrystallization of the reaction product mineral compositions. This is demonstrated by both the well-equilibrated textures (Figs. 2 and 3) and the general absence of compositional zoning in minerals.

An alternative hypothesis is that the magnetite component of the oxide mineral is progressively consumed with increasing metamorphism across the Chl-peridotite stability field (Fig. 1b), thereby passively enriching the oxide mineral in Cr_2_O_3_ so that it eventually becomes chromite. Such a scenario would either require loss of iron via aqueous fluid escape or transfer of iron into coexisting rock-forming silicates, such as olivine, orthopyroxene, and chlorite. Given that ferrous iron solubility in aqueous fluids is expected to be very low, and that of ferric iron even lower (e.g., Scholten et al 2019), fluid-mediated loss of iron from the rocks is not likely. Redistribution of iron from oxides into coexisting silicates could be monitored by inverse zoning in Mg# of olivine and orthopyroxene. No such zonation in Mg# of olivine and orthopyroxene is apparent from our data. However, it has been reported for Chl-harzburgite from Almirez that the first magnetite forming upon the antigorite-out reaction of moderately oxidized metaperidotites is Cr rich (up to 19 wt.% Cr_2_O_3_; Vieira Duarte et al 2021). Hence, it is well conceivable that chromite could form in hydrous metaperidotites containing low oxide modes directly at the antigorite-out reaction. Clearly, major heterogeneities exist in the extent of oxidation upon oceanic hydration and, consequently, of redox budget of the metaperidotites (e.g., Bretscher et al 2018). Notwithstanding such complications, the mineral compositions of Chr-Chl-peridotite, Chr-peridotite, and Grt-peridotite support the scenario of continuous consumption of chlorite along with redistribution of its element inventory but water into coexisting silicate and oxide minerals during prograde subduction to peak metamorphic conditions. This implies that continuous water liberation goes along with chlorite consumption, thus likely aiding in equilibration of the rock mineralogy to peak metamorphic conditions and a lowering of Δlog_10_*f*O_2_,_QFM_ due to the dilution of the magnetite component in spinel.

### Rock-buffered redox conditions at the Chl-out reactions

Redox buffer conditions in metaperidotites are established via the mineral assemblage olivine-orthopyroxene-magnetite. To apply the QFM equilibrium (quartz-fayalite-magnetite) to the peridotite assemblage, activities of quartz, fayalite, and magnetite are required. The magnetite and fayalite activities are determined by the compositions of the spinel and the olivine phases, respectively. The quartz activity is buffered by coexisting forsterite and enstatite in olivine and orthopyroxene. The link between the rock-buffered *f*O_2_ of the metaperidotites and the *f*O_2_ of the pure buffer assemblage is thus governed by reaction (7):

7 $${\text{6 Fayalite }} + {\text{ O}}_{{2}} = {\text{ 3 Ferrosilite }} + {\text{ 2 Magnetite}}{.}$$

Consequently, the composition of these minerals determines the extent of deviation in *f*O_2_ from the reference buffer conditions of QFM. This is the reason why all of the *f*O_2_ values pertaining to our metaperidotites are expressed as Δlog_10_*f*O_2_,_QFM_. In short, dilution of the FeO component in olivine increases Δlog_10_*f*O_2_,_QFM_ values, while dilution of Fe_2_O_3_ in magnetite via Cr_2_O_3_ and Al_2_O_3_ exchange reduces Δlog_10_*f*O_2_,_QFM_ values.

The above discussion implies that continuous chlorite decomposition beyond antigorite dehydration likely has consequences on the composition of rock oxides, which in turn affects the rock-buffered oxygen fugacity, because dilution of the iron content of the oxide mineral lowers the Δlog_10_*f*O_2_,_QFM_ of the rock buffer of a given sample.

The systematics of variably oxidized serpentinite protoliths have recently been modeled by Evans and Frost (2021), using two endmember cases, i.e., a “reduced” model with a reactive silicate bulk composition having a Mg# of 91.1 and an “oxidized” model with a reactive silicate bulk Mg# of 96.4, respectively. The results explored here are obtained from natural samples over a larger range of reactive silicate bulk Mg#s, characteristic for variable *f*O_2_ conditions imposed upon hydration on the ocean floor. From the previous discussion it follows that rock-buffered redox conditions can be assessed by the olivine Mg# combined with the magnetite component in spinel and (Fe, Cr) oxide modes.

### Rock buffered weakly to moderately oxidized conditions

Weakly to moderately oxidized conditions are found in most samples, where magnetite or chromite are the stable oxides, buffered by the mineral assemblage orthopyroxene + olivine + magnetite. Moderately oxidized conditions are observed in most Mag-Chl-peridotites, where oxide modes of 1–3 vol.% and olivine Mg# of 92–94 are observed. Indicative of extensive alteration at the seafloor, these samples show significant Ca loss, as shown in the Al_2_O_3_ vs. CaO plot (Fig. 7c). At moderately oxidized conditions, magnetite is the dominant stable oxide phase present, occurring at comparatively low P–T conditions (Alpe Albion and Val Cama). One Grt-peridotite (Cap18-03) from Alpe Capoli shows magnetite and ilmeno-hematite (Hem_63-74_Ilm_37-26_; Fig. 3h), coexisting with high Mg# olivine and orthopyroxene (94–95). These are consistent with *f*O_2_ estimates of + 1.5 log units above QFM given by the two-oxide Fe-Ti geothermobarometer of Ghiorso and Evans (2008), which suggests that moderately oxidizing conditions can occur at conditions higher that those required for chlorite dehydration at temperatures of ~ 800 °C (Lederer 2019).

Weakly oxidized conditions are observed in most Chr-Chl-peridotites, Chr-peridotites, and Grt-peridotites, characterized by low oxide modes (< 1 vol.%), and reactive silicate bulk Mg# (87–91). At these conditions, magnetite is the stable oxide present across the antigorite-out reaction (e.g., Cerro del Almirez, Vieira Duarte et al 2021; and Val Cama, this work), followed by transitions to chromite before complete chlorite-out reaction (e.g., Alpe Albion, Alpe Capoli, and Cima di Gagnone). Chromium enrichment and consequent Fe^3+^ decrease in magnetite contents shift the orthopyroxene-olivine-magnetite redox buffer to lower values. Therefore, chromite stability has the important implication to constrain the redox conditions to lower oxygen fugacity values prior to the last pulse of fluid release provided by the final chlorite-out reaction. Because of lower rock-buffered *f*O_2_, conditions for the transport of oxidized sulfur species in the fluids are not fulfilled. The results obtained here for weakly to moderately oxidized metaperidotites are thus in agreement with modeling results of Piccoli et al (2019), which document a drop in Δlog_10_fO_2_,_QFM_ of 3 log units upon chlorite dehydration. Moreover, Piccoli et al (2019) also reported an associated increase in the solubility of reduced sulfur species and a progressive replacement of magnetite by Cr-Al-bearing spinel.

Chromite stability extends to beyond complete prograde chlorite decomposition maintaining the rock-buffered oxygen fugacity at low values. For high pressures, this is confirmed by interstitial chromite grains in Grt-peridotites. Occasionally, Cr-spinel can also be found. At lower pressure conditions, transition of Al-chromite to Cr-spinel can be observed as in one Chr-Chl-peridotite (Alb18-03), where Cr-spinel occurs as interstitial grains, while Al-chromite is preserved as inclusion in orthopyroxene porphyroblasts.

Our results demonstrate that increased Cr and Al contents stabilize magnetite or chromite to even higher temperatures than pure magnetite. This Fe dilution effect drives the redox buffer to lower Δlog_10_fO_2_,_QFM_ values and thus extends the stability of magnetite and chromite toward temperature exceeding 800 °C. This is consistent with model implications by Piccoli et al (2019) and Evans and Frost (2021), which suggest that when including Cr in the model (Piccoli et al 2019) magnetite is lost in weakly oxidized samples just below 800 °C and the spinel component becomes more important.

### Evidence for rare HM-buffered redox conditions in Chl-peridotites

Near pure hematite (Hem_98±2_Ilm_02±2_) coexisting with magnetite (0.85 < XFe^3+^  < 0.95) was found as inclusions in peak olivine and orthopyroxene and in equilibrium with the peak mineral assemblage in one sample from Val Cama (PkCa-06; Fig. 3a, b), defining the Hem-Mag-Chl-peridotite metamorphic lithotype. Pure hematite represents the missing evidence for redox conditions at the HM buffer beyond the antigorite dehydration reaction at peak temperatures up to ca. 700 °C (compare also Evans and Frost 2021). Such highly oxidized metaperidotites coexist in the Val Cama ultramafic body with less oxidized metaperidotites containing Cr-magnetite and chromite but not hematite. The Hem-Mag-Chl-peridotite displays the highest Mg# measured in olivine (96) and orthopyroxene (95) of all the studied samples, the highest reactive silicate bulk Mg# of 95.2, as well as one of the highest oxide modes (2.6 vol.% / 4 wt.%) from all the Chl-peridotites and Grt-peridotites. Coexistence of hematite and magnetite constrains the rock-buffered oxygen fugacity to values between 3 and 4 log units above QFM, for a pressure range of between 1 and 2 GPa for Val Cama. Our findings are consistent with experimental results of Maurice et al (2020), obtained by dehydration experiments of natural Atg-serpentinite with 5.5 wt.% magnetite in a multi-anvil apparatus, which reported hematite and magnetite coexisting with peak olivine and orthopyroxene with high Mg# (94–97). The estimated Δlog_10_fO_2_,_QFM_ of our Hem-Mag-Chl-peridotite is significantly higher than values calculated for the moderately oxidized sample Cap18-03 (+ 1.5 Δlog_10_fO_2_,_QFM_) that contains Hem_63-74_Ilm_37-26_, or the maximum Δlog_10_fO_2_,_QFM_ of + 0.5 to + 1 for common harzburgite compositions (Piccoli et al 2019).

### Sulfide stability and paragenesis

Pentlandite and pyrrhotite are present in Chr-Chl-peridotites and some Grt-peridotites, while Hem-Mag-Chl-peridotites and Mag-Chl-peridotites do not contain sulfides. Pyrite was never observed. Highly variable sulfide modes in our metaperidotite samples are possibly due to (i) sulfur loss to dehydration fluids, (ii) variable sulfur contents inherited from partial melting and potential melt–rock reaction prior to oceanic serpentinization, and (iii) initial sulfur depletion or enrichment along with oceanic serpentinization or upon progressive subduction, or any combination thereof. Two important aspects need to be considered when assessing sulfur loss to dehydration fluids. The first is the capacity to mobilize sulfur in the fluid, which relates to sulfur solubility. The second is the fluid sulfur speciation that can be expected to affect sulfur solubility in the dehydration fluid. Sulfur speciation and solubility are both influenced by pressure, temperature, and the rock-buffered *f*O_2_ (e.g., Piccoli et al 2019; Evans and Frost 2021) that is controlled by the redox buffer of the hydrous metaperidotites.

**Fig. 11:**
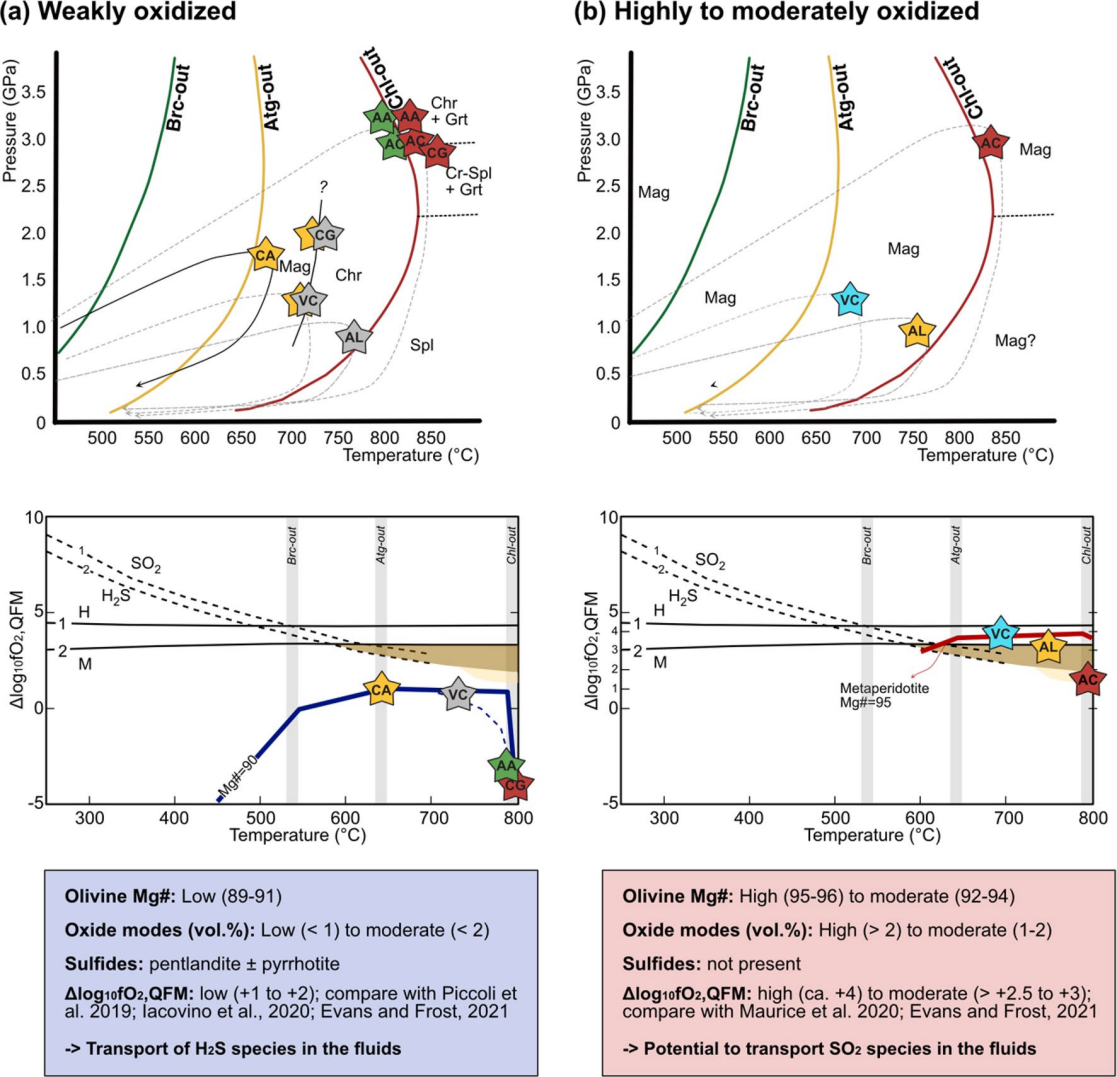
Summary figure showing oxide stability for a given sample locality in a pressure–temperature diagram for **a** weakly oxidized and for **b** highly to moderately oxidized metaperidotites. The dashed gray P–T paths are only indicative because the detailed P–T–t paths (notably between peak P and T) are not well constrained. Approximate fO_2_ conditions for a given sample locality (AA, Alpe Arami; AC, Alpe Capoli; AL, Alpe Albion; VC, Val Cama; CG, Cima di Gagnone; CA, Cerro del Almirez) are indicated in the lower diagrams, showing ∆log_10_fO_2,QFM_ vs. temperature (modified from Evans and Frost 2021). The solid blue line represents the modeled redox evolution for a metaperidotite with a reactive Mg# of 90 (Piccoli et al. 2019). The solid red line represents experimental redox evolution for a metaperidotite with a reactive Mg# of 95 containing hematite and magnetite (Maurice et al. 2020). In addition, the position of the hematite-magnetite (HM) buffer and the H_2_S-SO_2_ equal activity lines (SSO) are also represented for 1 and 2 GPa (Evans and Frost 2021). The yellow field thus represent the area below the HM but above the SSO line, where conditions for the formation of oxidized sulfur species are possible in the QFM-buffered domain at 2 GPa (darker yellow), which is expected to shift to lower fO_2_ at higher pressures (brighter yellow)

Figure 11 illustrates the modeled trend of the SO_2_-H_2_S equal activity line (i.e., where concentrations of SO_2_ and H_2_S are predicted to be equal; SSO) relative to the hematite-magnetite buffer in a plot of temperature versus ∆log_10_fO_2,QFM_. For the common weakly oxidized case (Fig. 11a), the evolution of rock-buffered ∆log_10_fO_2,QFM_ does not intersect the SSO line at antigorite dehydration; hence, any sulfur loss to the dehydration fluid is predicted to be present in its reduced form as H_2_S, and sulfur solubility in dehydration fluids is predicted to be low (Piccoli et al 2019; Evans and Frost 2021). Moreover, dehydration fluids also remain reduced across chlorite dehydration some 150 °C higher where sulfur solubility in fluid is predicted to increase by about a factor of 5 (Piccoli et al 2019). To a first order this is consistent with the presence of sulfides in weakly oxidized Chl-peridotites from Almirez (Vieira Duarte et al 2021; Piccoli et al 2019), and with very low sulfide modes (< 0.05 vol.%) in weakly oxidized Chr-Chl-peridotite, Chr-peridotites, and most Grt-peridotites from our study. However, it cannot account for the observation that all our Mag-Chl-peridotite and some weakly oxidized Grt-peridotite (e.g., CP16-10) samples do not contain sulfides.

For the case of highly oxidized rocks buffered at ∆log_10_fO_2,QFM_ of above ca. + 2.5 to + 3, the scenario is different (Fig. 11b). Here, the rock-buffered oxygen fugacity is predicted to be located above the SSO line at antigorite dehydration; hence, the stable fluid sulfur species is SO_2_ (Fig. 11). This certainly applies for rocks buffered by hematite-magnetite (our one sample PkCa-06), but it may also apply to moderately oxidized rocks just below the Hem-Mag buffer, i.e., buffered to between + 2.5 and + 3 log units above QFM. This domain, shaded yellow in Fig. 11, offers the opportunity for oxidized sulfur species, i.e., SO_2_, in the dehydration fluid without Hem-Mag buffer conditions established. Such conditions could have been attained in some of the Mag-Chl-peridotites (reactive silicate bulk Mg# between 92.9 and 93.5) and one Grt-peridotite (reactive silicate bulk Mg# of 94.5) from this study. With increasing temperature toward final chlorite-out, the corresponding ∆log_10_fO_2,QFM_ value is also reduced via increasing Cr-Al-Mg concentrations in the oxide mineral (see discussion above). As a consequence, the potential to forming SO_2_ in the coexisting aqueous fluid decreases. Even if SO_2_ would dominate in the aqueous fluid at these higher temperatures, modeling results of Piccoli et al (2019) predict a lower solubility and therefore less potential to mobilize redox budget in the fluid phase.

Initial bulk rock sulfur concentrations are most probably very variable. It has been proposed that during the first stages of oceanic serpentinization sulfides dissolve but can precipitate during the later stages by biotic or hydrothermal processes when the system reaches a fluid-buffered regime (Klein et al. 2009; Schwarzenbach et al 2012, 2018). Moreover, sulfur might be added upon progressive subduction via fluid infiltration, but no evidence for such a process has been found in our samples. Consequently, the sulfur concentration in peak metamorphic hydrous metaperidotites alone does not offer constraints on how much sulfur may have been lost during prograde to peak metamorphic dehydration reactions. Therefore, data on sulfur concentrations in fluid inclusion relics are needed to more reliably address the capacity to transporting sulfur in dehydration fluids.

Comparison of Ni contents in coexisting olivine and oxide minerals equilibrated at different P–T offers insights into the possible relation between initial heterogeneities acquired during serpentinization and metamorphic evolution of metaperidotites. At equilibrium, low Ni contents in olivine and magnetite can indicate the presence of a stable Ni–sulfide phase (e.g., Eq. (5) in Bretscher et al 2018; Kempf et al 2020; Vieira Duarte et al 2021). Most of the studied metaperidotites show olivine NiO contents between 0.30 and 0.60 wt.%, similar to concentrations reported for Cerro del Almirez (Bretscher et al 2018; compiled in Fig. 5). Figure 8d then illustrates that Ni/Fe ratios in the oxide correlate positively with olivine Mg# (Fig. 8d). This correlation suggests that initial heterogeneities caused by different extents in serpentinization might also play an important role on sulfur mobility during subduction; it is noted that sulfides are present in serpentinites from Zermatt with metamorphic olivine with high Mg# (Kempf et al 2020). Nevertheless, it is impossible to tell which processes cause the observed differences in Ni contents of oxide and the absence of sulfide itself. To test these possibilities and better constrain the mobility of S in moderately and highly oxidized metaperidotites, measurement of trace element concentrations in olivine fluid inclusions, as well as bulk rock S measurements, is required.

## Conclusions and implications for the redox conditions of the overall subducting hydrous ultramafic rocks

This study constrains the oxide mineralogy and geochemistry, in equilibrium with silicate mineral assemblages contained in compositionally heterogeneous metaperidotites, at different pressure and temperature conditions during Alpine subduction. Overall, oxide compositions depend both on the attained peak metamorphic conditions and on the extent of oxidation inherited from oceanic serpentinization. The latter can be approximated by the content of Fe-Cr-Al oxides in the metaperidotite and their mode, and by the Mg# of olivine and orthopyroxene in equilibrium. Two endmember scenarios can be envisaged: (i) Strongly oxidized conditions were observed in one metaperidotite containing pure hematite (Hem_>96_Ilm_<4_) and magnetite (Fig. 11b), coexisting with the very high olivine Mg# (95–96) and oxide modes (up to 4 vol.%). These conditions are consistent with *f*O_2_ at the HM buffer (Δlog_10_fO_2_,_QFM_ of + 3 to + 4; Maurice et al 2020) across the antigorite dehydration reaction. At these conditions oxidized sulfur species are predicted to be stable in the fluid phase (Evans and Frost 2021). (ii) At the other end of the range in rock oxidation state, weakly oxidized conditions (Fig. 11a) were observed in metaperidotites characterized by low oxide modes (< 1 vol.%) and coexisting low olivine Mg# (89–91). At these conditions, magnetite transitions to chromite and eventually to Cr-spinel at intermediate pressures with increasing temperature at about 700 °C (compare Piccoli et al 2019; Evans and Frost 2021). At these conditions, the system is buffered by the olivine-orthopyroxene-magnetite assemblage at Δlog_10_fO_2_,_QFM_ <  + 2.5 (Piccoli et al 2019; Iacovino et al 2020; Evans and Frost 2021), being always below the H_2_S-SO_2_ equilibrium line, i.e., at conditions consistent with reduced sulfur species in the dehydration fluid. Finally, scenarios at intermediate fO_2_ conditions can also be recognized. These correspond to moderately oxidized conditions (Fig. 11b), characterized by moderate oxide modes (1–2 vol.%) and olivine Mg# (92–94), always coexisting with magnetite. At these conditions, the rock buffered fugacity (between + 2.5 > Δlog_10_fO_2_,_QFM_ >  + 3 at the antigorite-out, or between + 1.5 > Δlog_10_fO_2_,_QFM_ >  + 2 at the chlorite-out; Evans and Frost 2021; this study) can be high enough to stabilize oxidized sulfur species in dehydration fluids (yellow regions in Fig. 11b). However, low sulfur fluid mobility at those conditions might reveal insufficient (compare Piccoli et al 2019) to justify the absence of sulfides.

Our work based on natural samples reveals that the redox budget of hydrous metaperidotite samples varies prominently and thus controls the oxidizing capacity of the fluids released upon antigorite and chlorite dehydration, in line with the concept presented by Evans and Frost (2021). Such bulk sample redox budget variations were inherited from serpentinite protoliths and most likely established upon oceanic serpentinization (compare Bretscher et al 2018). Serpentinites with high redox budget are thus more likely to mobilize oxidized sulfur species in the liberated antigorite and chlorite dehydration fluids during subduction when compared to the much more abundant, weakly oxidized serpentinites. In fact, it is not yet clear how representative the single highly oxidized sample (olivine Mg# > 95) from this study is for the global subduction cycle. Highly oxidized serpentinites are expected to occur in some portions of the ocean-continent transition settings (Evans and Frost 2021). Moreover, high Mg# observed in antigorite and olivine in antigorite-peridotites from Cerro del Almirez (Bretscher et al 2018) and Zermatt (Kempf et al 2020) suggest that such conditions could occur if these rocks would have crossed the antigorite-out reaction. Without excluding the possibility of some transfer of redox budget to the dehydration fluids, the stability of magnetite beyond the chlorite-out reaction in moderately to highly oxidized samples implies that at least part of the redox budget inherited from ocean floor hydration is transported to greater depths at T > 800 °C.

Finally, specific enrichments in Fe, Sc, Ni, and Cu are observed in oxide minerals in the highly oxidized samples relative to the weakly oxidized metaperidotites, which in turn show enrichments in Cr, Al, Zn, Co, Ga, Zr, and Nb. Significant enrichments in fluid-mobile and redox-sensitive elements (e.g., Fe, Cr, Zn, V, Ga, Ti, Sn, Mo, Pb, W, and U) are hosted in oxide minerals in both highly to weakly oxidized metaperidotites that can be transported within the subducting slab to depths exceeding antigorite- and chlorite-out reactions. 

### Supplementary Information

Below is the link to the electronic supplementary material.

Supplementary file1 (DOCX 2422 KB)

Supplementary file2 (DOCX 2956 KB)

Supplementary file3 (DOCX 6203 KB)

Supplementary file4 (DOCX 3832 KB)

Supplementary file5 (DOCX 19 KB)

Supplementary file6 (XLSX 11 KB)

Supplementary file7 (XLSX 194 KB)

## Data Availability

All underlying data related to this work is available at 10.5281/zenodo.7516492.
