# Supplementary material for: Oxide-silicate petrology and geochemistry of subducted hydrous ultramafic rocks beyond antigorite dehydration (Central Alps, Switzerland)
Source: Contrib Mineral Petrol. 2023 Aug 16;178(9):60. doi: 10.1007/s00410-023-02032-w (PMC11008075; doi:10.1007/s00410-023-02032-w)

**Supplementary Figure S1 – Sulfide petrography**

Microphotographs of the sulfide minerals present in the studied sub-lithologies, acquired by (a) BSE microscopy and (b,c) reflected light microscopy. (a) Pentlandite in the Chr-peridotites overgrown by retrograde chlorite. (b, c) Pentlandite and pyrrhotite inclusions in (b) orthopyroxene and (c) garnet in the Grt-peridotites. Inset in (c) was obtained by BSE microscopy.


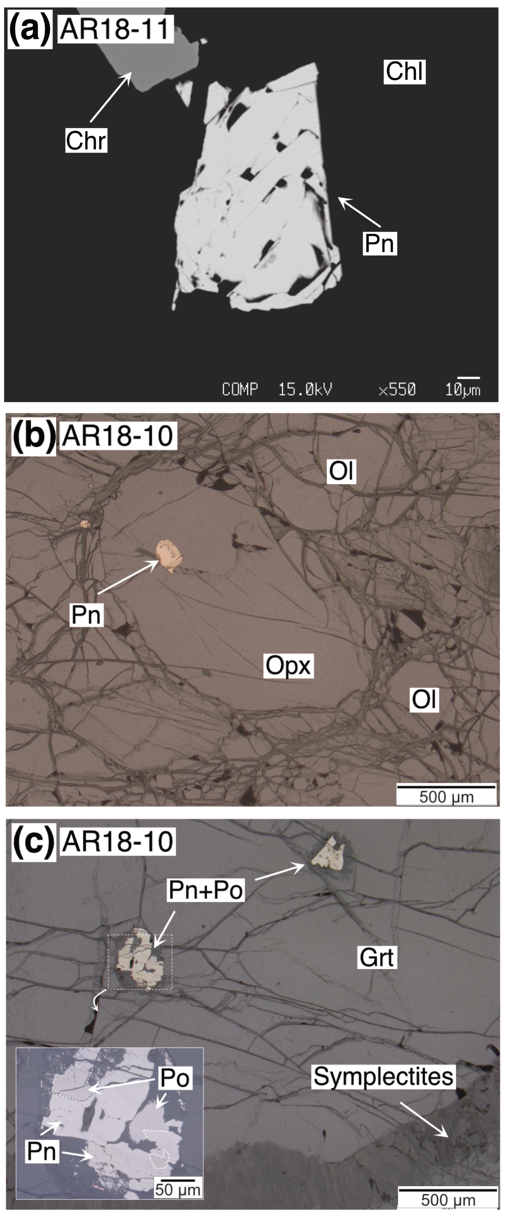

Supplement: Supplementary file 1 — Supplementary file1 (DOCX 2422 KB) [file 410_2023_2032_MOESM1_ESM.docx]
