# Supplementary material for: Oxide-silicate petrology and geochemistry of subducted hydrous ultramafic rocks beyond antigorite dehydration (Central Alps, Switzerland)
Source: Contrib Mineral Petrol. 2023 Aug 16;178(9):60. doi: 10.1007/s00410-023-02032-w (PMC11008075; doi:10.1007/s00410-023-02032-w)

**Supplementary Figure S2 – Retrogression textures**

Textures of retrograde Grt-peridotite Cap18-03 from Alpe Capoli. (a) Lizardite/chrysotile along fractures in olivine and brownish patches with symplectites of amphibole and green Mg-Al-spinel. (b) Zoom-in on symplectites of amphibole and spinel, forming straight boundaries with silicate minerals (olivine, orthopyroxene, amphibole). These were interpreted to represent former garnet crystals, matched by bulk EDS measurements. Pyroxenite sample CP16-09 from Alpe Capoli (not used in this work) shows symplectites forming at the rims of garnet crystals. These symplectites show a general depletion in FeO and enrichment in CaO relative to preserved garnet cores. Symplectites in CP16-09 are similar in composition to those found in CP16-10 and Cap18-03, despite slight differences in MgO, FeO, and CaO, which likely reflect the influence of bulk composition. We can thus safely interpret these symplectites from Alpe Capoli to have been formed at the expense of garnet during decompression.


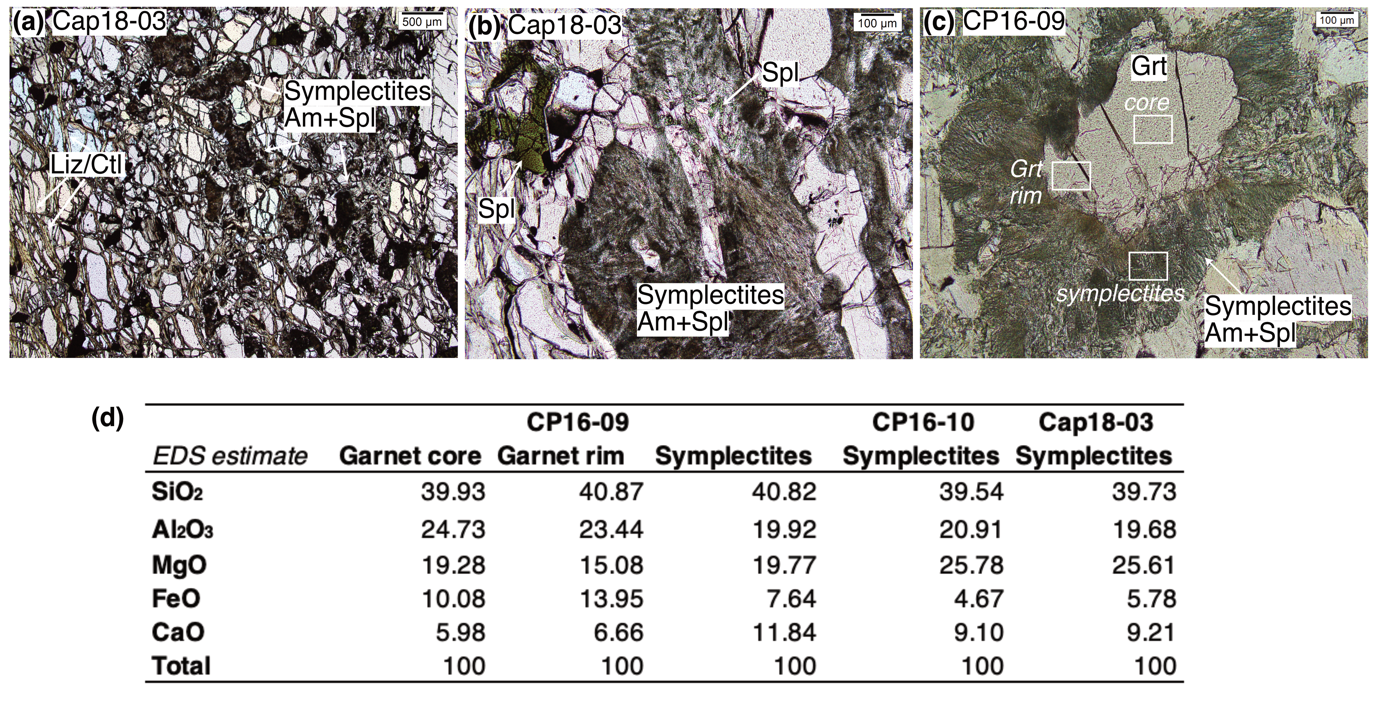

Supplement: Supplementary file 2 — Supplementary file2 (DOCX 2956 KB) [file 410_2023_2032_MOESM2_ESM.docx]
