# Supplementary material for: Oxide-silicate petrology and geochemistry of subducted hydrous ultramafic rocks beyond antigorite dehydration (Central Alps, Switzerland)
Source: Contrib Mineral Petrol. 2023 Aug 16;178(9):60. doi: 10.1007/s00410-023-02032-w (PMC11008075; doi:10.1007/s00410-023-02032-w)

**Supplementary Figure S3 – Oxide LA-ICP-MS data**

Oxide major and trace element compositions acquired by LA-ICP-MS and normalized to primitive mantle (PM; Palme and O’Neill 2014). (a) Fe-Cr-Al oxides plotted by studied lithology, to which are overlain magnetite compositions from Chl-peridotites of Cerro del Almirez (grey field; Vieira Duarte et al. 2021). (b) Hematite and ilmenite compositions in comparison to Fe-Cr-Al oxides in all lithologies. (c) Hematite and magnetite compositions from the Hem-Mag-Chl-peridotite (PkCa06), and ilmeno-hematite in Grt-peridotite (Cap18-03) compared to ilmeno-hematite compositions in Chl-peridotite from Cerro del Almirez (grey field; Vieira Duarte et al. 2021).


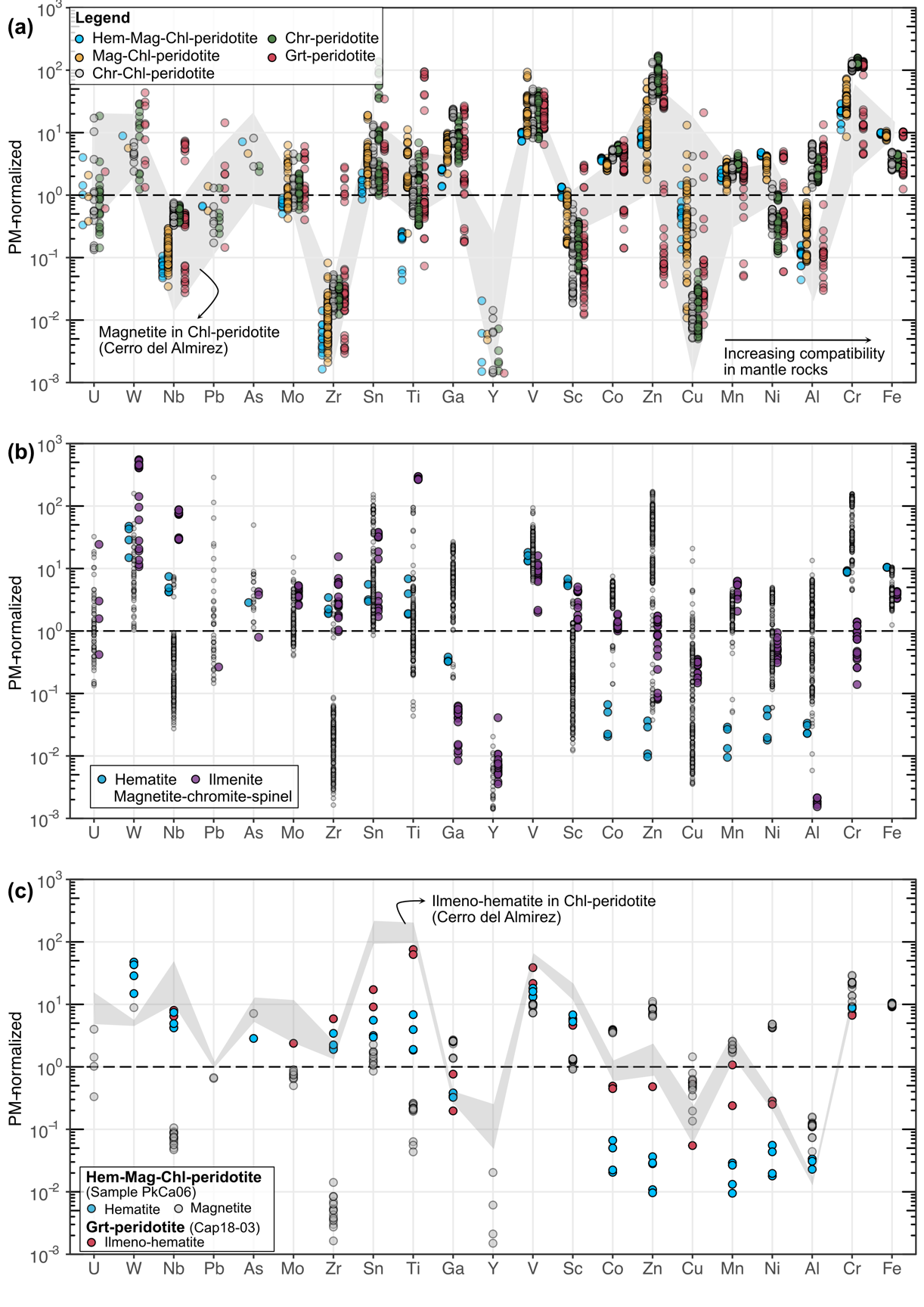

Supplement: Supplementary file 3 — Supplementary file3 (DOCX 6203 KB) [file 410_2023_2032_MOESM3_ESM.docx]
