# Supplementary material for: Oxide-silicate petrology and geochemistry of subducted hydrous ultramafic rocks beyond antigorite dehydration (Central Alps, Switzerland)
Source: Contrib Mineral Petrol. 2023 Aug 16;178(9):60. doi: 10.1007/s00410-023-02032-w (PMC11008075; doi:10.1007/s00410-023-02032-w)

**Supplementary Figure S4 – In-situ Al-enrichments in prograde silicates**

Averaged mineral Al_2_O_3_ concentrations obtained by EPMA for (a) orthopyroxene and (b) amphibole crystals, in the different metaperidotites. In (a) the composition of orthopyroxene porphyroblasts is represented for core (filled symbols) and rims (empty symbols), showing in some cases an increase in Al_2_O_3_ contents towards the rims. In (b) the composition of amphibole shows a clear increase in Al_2_O_3_ from magnetite-bearing to chromite-bearing lithologies.


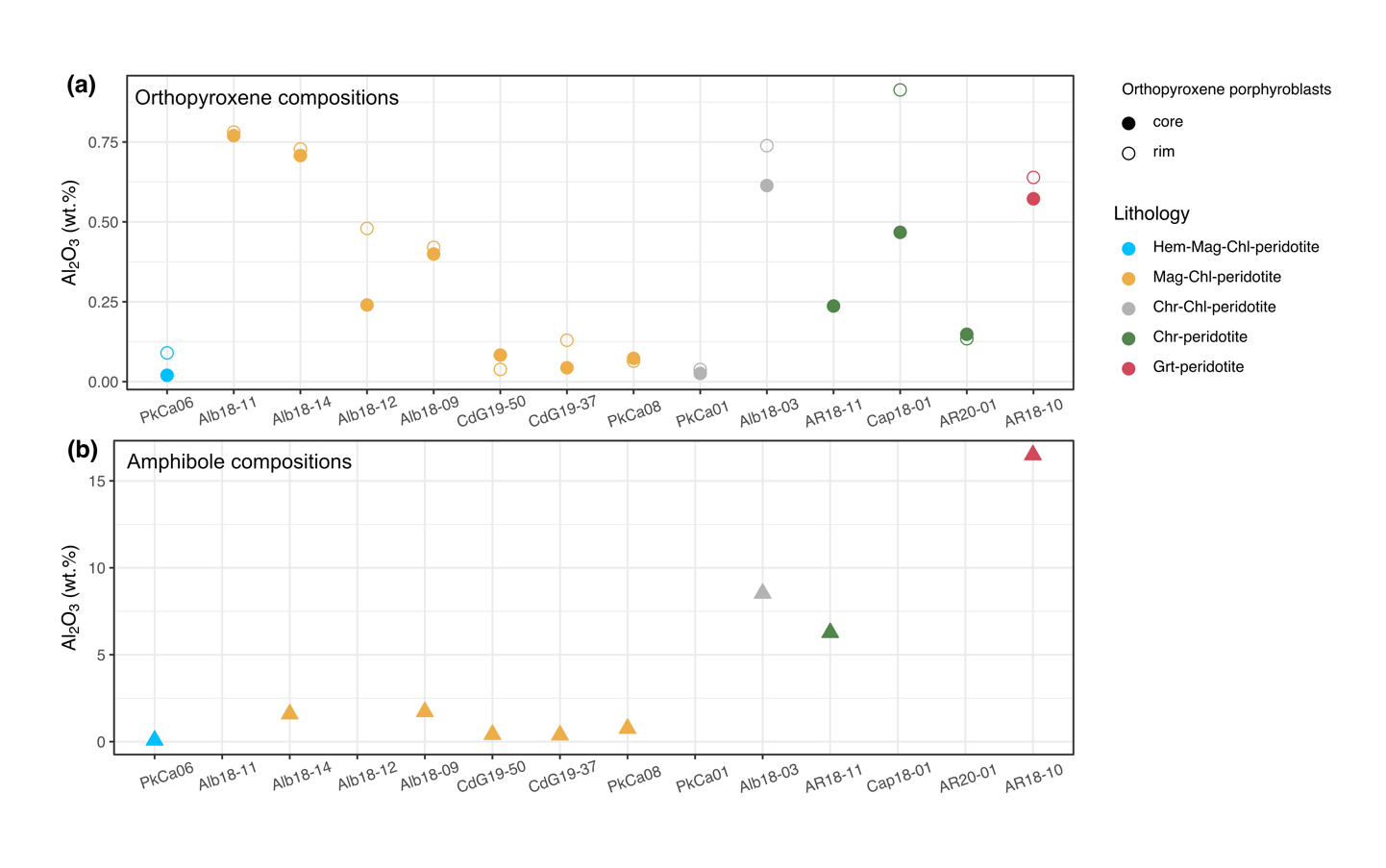

Supplement: Supplementary file 4 — Supplementary file4 (DOCX 3832 KB) [file 410_2023_2032_MOESM4_ESM.docx]
