# Supplementary material for: Oxide-silicate petrology and geochemistry of subducted hydrous ultramafic rocks beyond antigorite dehydration (Central Alps, Switzerland)
Source: Contrib Mineral Petrol. 2023 Aug 16;178(9):60. doi: 10.1007/s00410-023-02032-w (PMC11008075; doi:10.1007/s00410-023-02032-w)
Supplement: Supplementary file 5 — Supplementary file5 (DOCX 19 KB) [file 410_2023_2032_MOESM5_ESM.docx]

# Supplementary Information S1 – Methods

# EPMA major element analysis

In-situ major element abundances in silicate, oxide and sulfide minerals were determined by WDS using a JEOL JXA 8200 electron probe microanalyzer at the Institute of Geological Sciences of the University of Bern. Silicate minerals (olivine, ortho- and clinopyroxene, chlorite, serpentine, amphibole, and talc) were measured using an acceleration voltage of 15 kV, a probe current of 20 nA, and a beam diameter of 2 μm. Mass fractions of ten element oxides were calibrated using synthetic and natural standards: orthoclase (SiO_2_; K_2_O), forsterite (MgO), magnetite (FeO), anorthite (Al_2_O_3_; CaO), chromium-spinel (Cr_2_O_3_), pyrope (MnO), nickel oxide (NiO), rutile (TiO_2_), albite (Na_2_O), and tugtulite (Cl).

Oxide measurements were performed using an acceleration voltage of 15 kV, a probe current of 100 nA, with a beam diameter of 10 μm to prevent sample overheating. High probe currents allows decreasing the limit of detection of minor elements (Al, Ti, Zn, Cr, Ni; Dupuis and Beaudoin 2011). A beam size of 2 μm and probe current of 20 nA was used for ~5-10 μm large oxide inclusions in porphyroblasts of olivine, orthopyroxene, and chlorite. The mass fractions of seven element oxides were calibrated using synthetic and natural standards: almandine/magnetite (FeO), chromium spinel (Cr_2_O_3_), anorthite/spinel (Al_2_O_3_), forsterite (MgO), tephroite/pyrope (MnO), nickel oxide (NiO), zinc oxide (ZnO), rutile (TiO_2_). Ferric iron in magnetite was calculated by charge balance from stoichiometric compositions. Reintegration of ilmeno-hematite solid solution grains was done by recalculating the composition by considering 10 and 18 vol.% of ilmenite rims as estimated by image analysis.

Element maps were acquired for oxides in 3 samples (Mag-Chl-peridotites Alb18-11 and CdG19-37, and Grt-peridotite Cap18-03), following the analytical procedure given in Lanari et al (2014). The analytical conditions were 15 kV accelerating voltage, 100 nA probe current and dwell times of 150-200 ms. The sizes of the maps and dwell times are displayed in Table S1 for each sample. Elemental maps include Mg, Fe, Al, Ti, Cr, Ni, and Mn acquired in WDS mode, and V and Zn acquired in energy-dispersive spectrometry (EDS) mode. Data processing and calibration were performed using XMapTools 3.4.1. (Lanari et al 2014, 2019). The internal analytical standardization was performed using in situ spot analyses acquired on the same area.

Sulfide measurements were performed at similar conditions as for the oxide inclusions in silicate minerals (i.e., 15 kV acceleration voltage, 20 nA probe current, and 2 µm beam size). In this case, mass fractions of six elements were calibrated using synthetic and natural standards: pyrite (Fe, S), pentlandite (Ni, Co), galena (Pb), chalcopyrite (Cu).

| Lithology | Sample | Dimensions (µm) | Dwell time (ms) |
| --- | --- | --- | --- |
| Mag-Chl-peridotite | Alb18-11 | 500 x 500 | 200 |
| Mag-Chl-peridotite | CdG19-37 | 450 x 350 | 150 |
| Grt-peridotite | Cap18-03 | 900 x 600 | 200 |

**Table S1.** EPMA acquisition details of compositional maps acquired for oxide minerals.

**Reference**

Dupuis C, Beaudoin G (2011). Discriminant diagrams for iron oxide trace element fingerprinting of mineral deposit types. Mineralium Deposita 46, 319–335.

Lanari P, Vidal O, De Andrade V, Dubacq B, Lewin E, Grosch EG, Schwartz S (2014). XMapTools: A MATLAB © - based program for electron microprobe X-ray image processing and geothermobarometry. Computers and Geosciences 62: 227–240.

Lanari P, Vho A, Bovay T, Airaghi L, Centrella S (2019). Quantitative compositional mapping of mineral phases by electron probe micro-analyser. In: Ferrero S, Lanari P, Gonçalves P, Grosch EG (eds) Metamorphic Geology: Microscale to mountain belts, Geological Society of London Special Publication 478: 39–63.
